# Mitochondrial nucleoid remodeling and biogenesis are regulated by the p53-p21^WAF1^-PKCζ pathway in p16^INK4a^-silenced cells

**DOI:** 10.18632/aging.103029

**Published:** 2020-04-24

**Authors:** Yun Yeong Lee, Yeon Seung Choi, Do Wan Kim, Jae Youn Cheong, Kye Yong Song, Min Sook Ryu, In Kyoung Lim

**Affiliations:** 1 Department of Biochemistry and Molecular Biology, Ajou University School of Medicine, Suwon 16499, Korea; 2 Department of Biomedical Sciences, The Graduate School, Ajou University, Suwon 16499, Korea; 3 Department of Otolaryngology, Ajou University School of Medicine, Suwon 16499, Korea; 4 Omics Center, Ajou University School of Medicine, Suwon 16499, Korea; 5 Department of Gastroenterology, Ajou University of Medicine, Suwon 16499, Korea; 6 Department of Pathology, Chung-Ang University College of Medicine, Seoul 156-756, Korea

**Keywords:** senescence, p53-p21-PKCζ activation, p16INK4a silence, mitochondria, nucleoid remodeling

## Abstract

Mitochondrial dysfunction is linked to age-related senescence phenotypes. We report here the pathway increasing nucleoid remodeling and biogenesis in mitochondria during the senescence of foreskin human diploid fibroblasts (fs-HDF) and WI-38 cells. Replicative senescence in fs-HDF cells increased mitochondrial nucleoid remodeling as indicated by 5-bromo-2'-deoxyuridine (BrdU) incorporation and mitochondrial transcription factor A (TFAM) expression in enlarged and fused mitochondria. Mitochondrial nucleoid remodeling was accompanied by mitochondrial biogenesis in old cells, and the expression levels of OXPHOS complex-I, -IV and -V subunits, PGC-1α and NRF1 were greatly increased compared to young cells. Activated protein kinase C zeta (PKCζ) increased mitochondrial activity and expressed phenotypes of delayed senescence in fs-HDF cells, but not in WI-38 cells. The findings were reproduced in the doxorubicin-induced senescence of young fs-HDF and WI-38 cells *via* the PKCζ-LKB1-AMPK signaling pathway, which was regulated by the p53-p21^WAF1^ pathway when p16^INK4a^ was silenced. The signaling enhanced PGC-1α-NRF1-TFAM axis in mitochondria, which was demonstrated by Ingenuity Pathway Analysis of young and old fs-HDF cells. Activation of the p53-p21^WAF1^ pathway and silencing of p16^INK4a^ are responsible for mitochondrial reprogramming in senescent cells, which may be a compensatory mechanism to promote cell survival under senescence stress.

## INTRODUCTION

Cellular senescence is involved in important biological processes, *e.g.*, development, aging, and tumorigenesis, and is induced by activation of the p53-21^WAF1^ or p16^INK4a^-pRB axis [1]. Repression of p16^INK4a^ expression delays senescence and regulates the replicative senescence phenotype [2]. Expression of p16^INK4a^ is regulated by complex pathways involving lymphoid-specific helicase (Lsh), which binds to the p16^INK4a^ promoter and creates a repressive chromatin structure by recruiting HDAC1 [3].

The free-radical theory of aging [4] proposes that progressive accumulation of mitochondrial dysfunction in aged cells might be due to increased production of reactive oxygen species (ROS). Not only in senescent cells, but also *in vivo* skeletal muscle, mitochondrial bioenergetics and mitochondrial membrane potential differences (Δψm) are significantly impaired in aged animals [5], providing a cellular basis for aging-related mitochondrial defects. Oxidative damage to proteins and mitochondrial DNA (mtDNA) is associated with accumulation of mtDNA mutations [6, 7]. However, mitochondrial oxidative metabolism is upregulated in senescent cells as a metabolic requirement [8, 9]. Partial uncoupling of oxidative phosphorylation in mitochondria has been reported in senescent fibroblasts [10], and BRAF^V600E^- and RAS^G12V^-induced senescence upregulates the tricarboxylic acid (TCA) cycle and respiration by activating pyruvate dehydrogenase [9]. The mechanism underlying discrepant mitochondrial activity in senescent cells needs to be investigated.

mtDNA is packaged into aggregates with proteins, known as nucleoids [11]. Multicopy mtDNAs are assembled with DNA-binding proteins, such as mitochondrial transcription factor A (TFAM), in the mammalian mitochondria to form nucleoid structures [12]. Several copies of mtDNA are bound to nucleoid proteins, such as mitochondrial single-stranded DNA-binding protein (mtSSB), TFAM, and DNA-polymerase gamma (POLγ) [13, 14]. Nucleoids can be remodeled and adopt an enlarged punctate structure to protect mtDNA against damage induced by anticancer DNA-intercalating agents. These effects are mediated by the DNA damage response *via* ATM/p53 activation [15]. TFAM is a transcriptional activator in mitochondria for the mitochondrial-encoding OXPHOS complex genes and is a fundamental component of the basal mtDNA transcription machinery [16, 17]. Disruption of the TFAM gene in mice leads to embryonic lethality with mtDNA loss [18], whereas increased TFAM expression results in multiple copies of mtDNA [19]. Confocal microscopic analysis revealed colocalization of a number of nucleoid proteins with mtDNA. Thus, the association of mtDNA with TFAM, other proteins, and BrdU incorporation is essential in the nucleoid to retain mtDNA [13, 14].

Unexpectedly, we observed marked incorporation of BrdU into mitochondria in old, but not young, fs-HDF cells, together with increased expression of mtDNA genes and TFAM, implying mitochondrial nucleoid remodeling. The phenomenon was accompanied by mitochondrial biogenesis, regulated by PGC-1α and NRF1 expression *via* activation of LKB1 and AMPK, which are downstream of PKCζ, in old fs-HDF cells. Protein kinase C zeta (PKCζ), an atypical PKC (aPKC) subfamily, has been reported as a key regulator of the intracellular signaling pathways induced by various extracellular stimuli [20]. The activated PKCζ regulates AMPK activity by direct phosphorylation of LKB1 on Ser^428^ residue under conditions of ROS stress and energy depletion [21, 22]. Moreover, expression of PKCζ is most abundant in fs-HDF cells [23]. Despite the various cellular functions of PKCζ, however, its role in regulation of cellular senescence is not yet reported. Thus, we were tempted to investigate its role in mitochondrial remodeling in senescence of human fibroblasts, and found that mitochondrial nucleoid remodeling and biogenesis were regulated by activation of the p53-p21^WAF1^ pathway in p16^INK4a^-silenced cells. We suggest that PKCζ plays a key role in regulating LKB1-dependent AMPK activation in senescent cells by regulating mitochondrial nucleoid remodeling at the downstream of the p53-p21^WAF1^ pathway. Our data imply that mitochondrial reprogramming may delay senescence and promote survival of the p16^INK4a^-silenced cells.

## RESULTS

### Replicative senescence of fs-HDF cells leads to mitochondrial nucleoid remodeling and biogenesis

**Figure 1:**
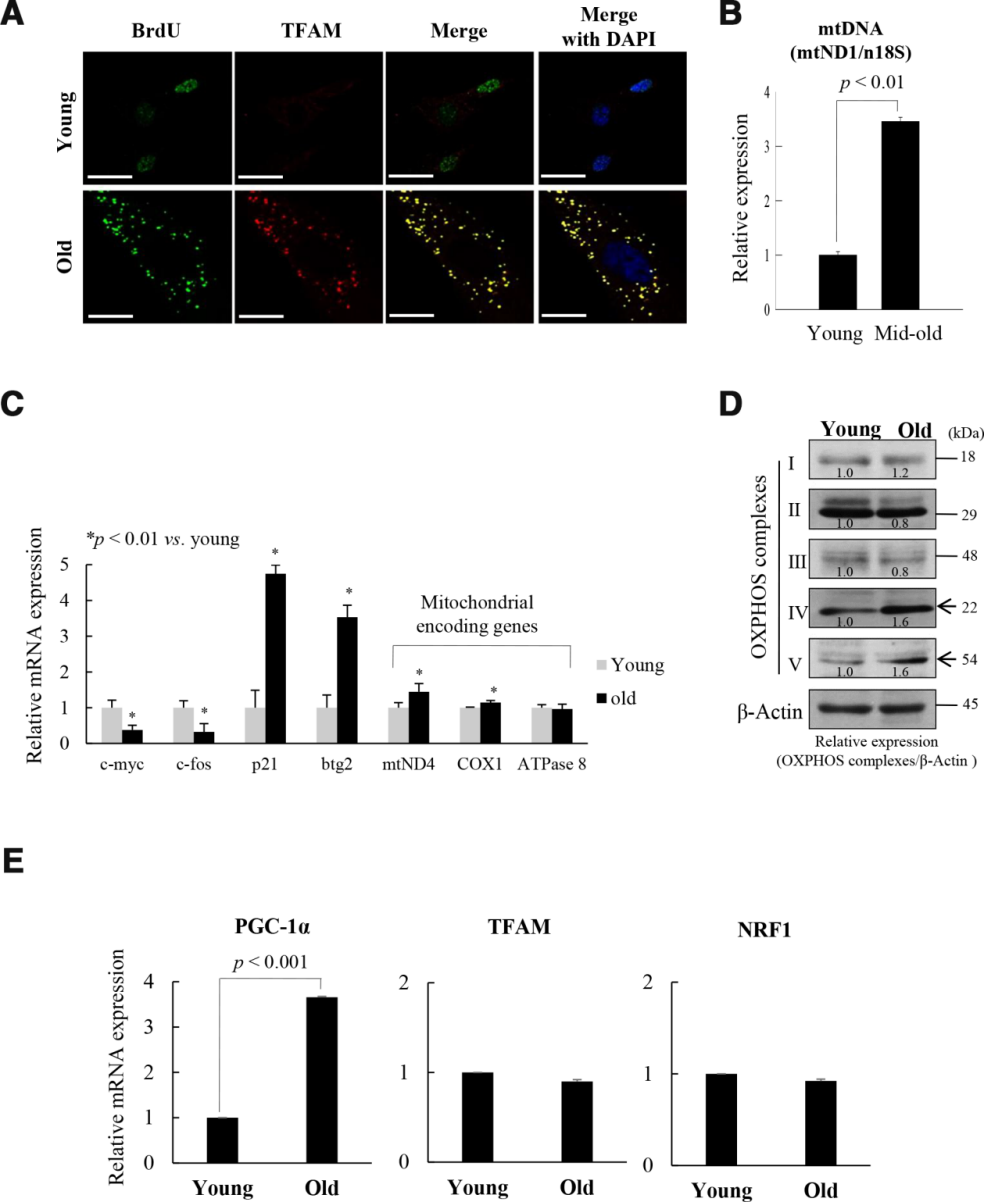
**Replicative senescence of foreskin human diploid fibroblast (fs-HDF) is accompanied by mitochondrial nucleoid remodeling and biogenesis.** (**A**) Immunofluorescence staining of BrdU incorporation into young and old fs-HDF cells. BrdU (green) and mitochondrial transcription factor A (TFAM; red) were observed by confocal microscopy. Nuclei were stained with DAPI (blue). Scale bars, 25 μm. (**B**) RT-qPCR analysis of the mitochondrial DNA (mtDNA) level, normalized to that of nDNA. (**C**) RT-qPCR analysis of the expression of nuclear- and mitochondrial-encoded genes. The expression levels of mitochondrial OXPHOS complex-I, -IV, and -V; c-myc and c-fos (markers of proliferation), and p21^WAF1^ and btg2 (markers of cell-cycle arrest) were analyzed. (**D**) Immunoblot analysis of OXPHOS complex I–V. β-actin was used as the loading control. Band intensity was quantified using ImageJ software (NIH, Bethesda, MD, USA) and normalized to that of β-actin. (**E**) Relative mRNA levels of PGC-1α, TFAM, and NRF1 by RT-qPCR. Data are means ± standard deviation (SD) of three independent experiments per group.

Mitochondrial nucleoids are composed of mtDNA and TFAM [14], and co-localized with mtSSB [13]. BrdU incorporation was associated with TFAM expression in the cytoplasm of old fs-HDF cells (doubling time [DT]: 2 weeks), but in the nuclei of young cells (Figure 1A). The data imply that mitochondrial biogenesis activity was higher in the old cells than in the young cells. As confirmation of mitochondrial activity in old cells, immunofluorescence microscopy showed that mtDNA, mtDNA polymerase γ (POL-γ), and Tom20 expression cooccurred with BrdU incorporation outside the nucleus (Supplementary Figure 1). The amount of mtDNA was threefold higher in senescent (DT: 1 week) cells compared to young cells (Figure 1B), further implying remodeling of mitochondrial nucleoids in the senescent cells. However, the amount and the activity of POL-γ did not significantly differ between the young and old cells (Supplementary Figure 2). These data are in accordance with previous reports that the activity of POL-γ is constant throughout the lifespan of cells [24], although 3'-5' exonuclease activity is altered during cellular senescence [25]. Both mRNA (Figure 1C) and protein (Figure 1D, Supplementary Figure 1C) expressions of the mitochondrial OXPHOS subunit genes, mtND4 (complex I), COX1 (complex IV), and complex V were significantly higher in old cells than in young cells, whereas c-Fos and c-Myc expression was downregulated in contrast to upregulation of p21^WAF1^ and btg2 in old cells.

To evaluate the role of mitochondrial biogenesis in the replicative senescence of fs-HDF cells, the expression of the TFAM regulator, PGC-1α, was assayed by RT-qPCR and immunoprecipitation-immunoblot analyses. Expression of PGC-1α was significantly higher in the old cells than in the young cells (Figure 1E, *p < 0.001* and Supplementary Figure 3A, 3B), implying that mitochondrial biogenesis was activated in the old cells. This is supported by a report that PGC-1α is a central integrative regulator in the transcriptional regulatory cascade of the mitochondrial biogenic response [26]. To evaluate the replicative senescence of fs-HDF cells further, mitochondrial alteration was examined by electron microscopy; significant elongation and fusion in abnormal shapes (Supplementary Figure 4A), and increased length of mitochondrial x-axis and y-axis (Supplementary Figure 4B, 4C, *p < 0.05 vs.* young cells). The expression of an elongation factor, OPA1, was higher in old cells than in young cells (Supplementary Figure 4D, *p < 0.01*), whereas number of mitochondria was not different between the young and old cells (Supplementary Figure 4E). In addition, the cells showed loss of mitochondrial Δψm; following staining with JC-1, the enlarged old cells, but not the young cells, showed green fluorescence (Supplementary Figure 4F, 4G). The ROS level was significantly higher, and the ATP content was significantly lower, in the old cells (Supplementary Figure 4H, 4I). These findings imply that replicative senescence of fs-HDF cells involves mitochondrial nucleoid remodeling and biogenesis, despite the altered mitochondrial morphology.

### PKCζ regulates mitochondrial reprogramming in fs-HDF old cells

**Figure 2:**
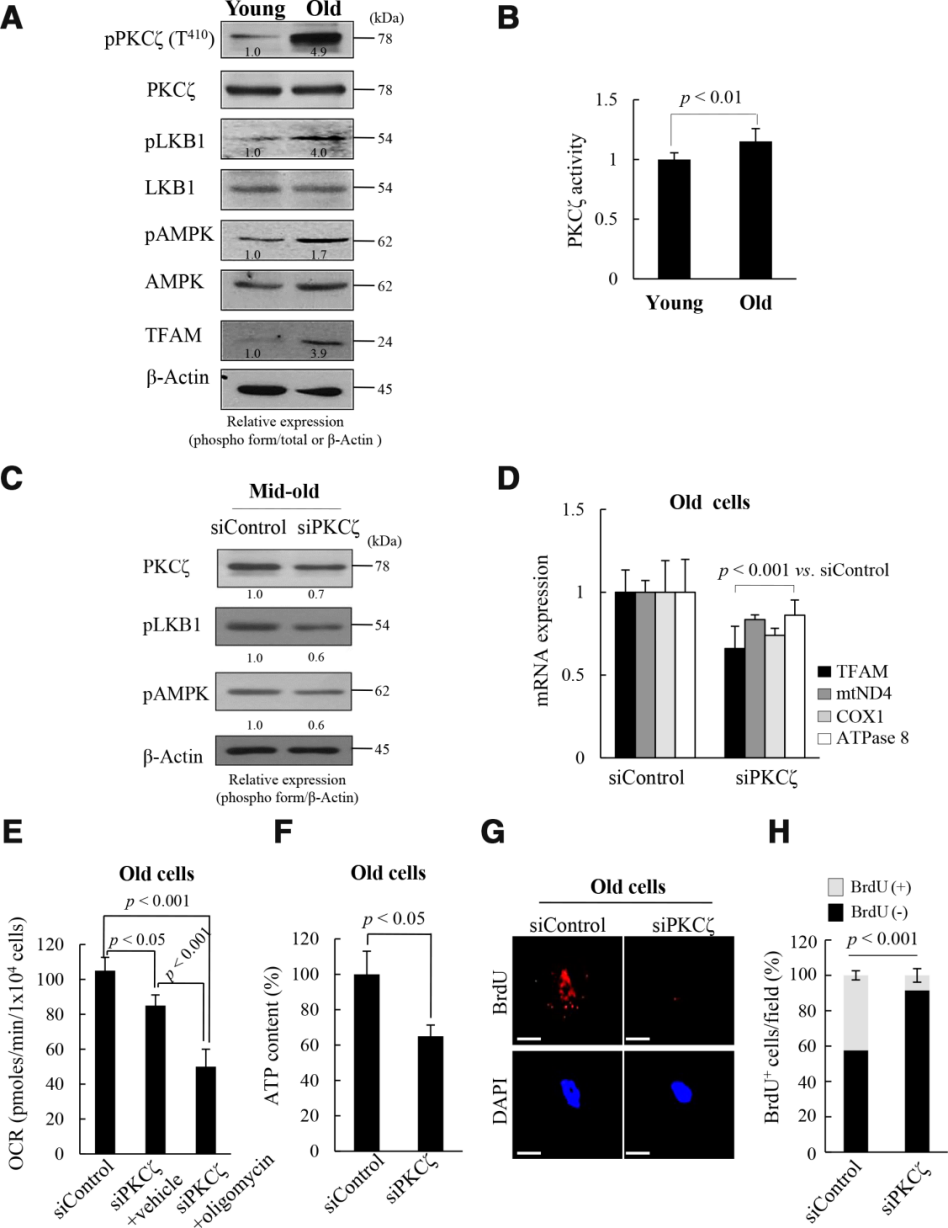
**Protein kinase C zeta (PKCζ) regulates mitochondrial reprogramming in old fs-HDF cells.** (**A**) Immunoblot of PKCζ, LKB1, AMPK, and TFAM in young and old cells. (**B**) PKCζ was purified from young and old cells by immunoprecipitation (IP) and subjected to *in vitro* kinase assay using a PKC kit. (**C**) Mid-old fs-HDF cells were transfected with siRNAs-PKCζ and subjected to immunoblot analysis. Band intensity was quantified using ImageJ software and normalized to β-actin. (**D**) Old fs-HDF cells transfected with siPKCζ were subjected to RT-qPCR to measure the expression of TFAM and the mitochondrial complex-I (ND4), -IV (COX1), and -V (ATPase8) subunits. Data were normalized to the siControl-transfected cells. (**E**) Old fs-HDF cells transfected with siPKCζ were treated with or without oligomycin (10 μM) for 1 h and the oxygen consumption rate (OCR; pmol/min/1 × 10^4^ cells) was compared to that of siControl-transfected cells. Note significant inhibition of OCR by knockdown of PKCζ expression. It was more downregulated by oligomycin cotreatment. (**F**) ATP levels in old cells transfected with siPKCζ. (**G**) BrdU incorporation in old cells transfected with siControl or siPKCζ. Nuclei were stained with DAPI (blue). Scale bars, 10 μm. (**H**) BrdU incorporation in mitochondria was quantified. Confocal microscope images were captured and counted at least 200 cells using ImageJ software (n=10 images/group). Data are means ± SD of two independent experiments per group. Student’s *t*-test or one-way ANOVA followed by Tukey HSD *post hoc* test.

To explore the upstream signals that regulate mitochondrial nucleoid remodeling during replicative senescence of fs-HDF cells, the LKB1-AMPK signal pathway and its upstream kinase were evaluated in the presence of high ROS conditions. The activities of LKB1 and AMPK were higher in the old cells than in the young cells, as was expression of TFAM (Figure 2A). Moreover, PKCζ activity was significantly higher in the old than the young (Figure 2A, 2B, *p < 0.01*). When PKCζ expression was knocked-down by transfecting senescent cells with short interfering RNAs (siRNAs)-PKCζ, phosphorylation of LKB1 and AMPK was reduced by up to 40% (Figure 2C), and expression of TFAM and its target genes, ND4, COX1 and ATPase 8, decreased in the old cells (Figure 2D, *p < 0.001*). Knockdown of PKCζ also reduced the mitochondrial oxygen consumption rate (OCR; Figure 2E, *p < 0.05*) and ATP content (Figure 2F, *p < 0.05*) compared to siControl-transfected cells. Moreover, oligomycin cotreatment further inhibited the mitochondrial OCR than the siRNAs-PKCζ with vehicle treatment (Figure 2E, *p < 0.001 vs.* siControl and siPKCζ), indicating that the signal is transmitted *via* the PKCζ-LKB1-AMPK axis. Indeed, BrdU incorporation into mitochondrial nucleoids was downregulated by transfecting old cells (DT: 3 weeks) with siRNAs-PKCζ compared to the siControl (Figure 2G, 2H, *p < 0.001*). When the role of PKCζ in mitochondrial BrdU incorporation was assessed using a PKCζ inhibitor, mitochondrial BrdU incorporation and activations of LKB1 and AMPK were found to be reduced in the old cells (Supplementary Figure 5). The data imply that mitochondrial nucleoid remodeling and biogenesis are regulated *via* the PKCζ-LKB1-AMPK signaling pathway in replicative senescence of fs-HDF cells.

### Mitochondrial reprogramming is recapitulated in the induced senescence of fs-HDF cells

To explore whether the above signaling pathway regulates mitochondrial reprogramming during senescence, young fs-HDF cells were treated with doxorubicin (Doxo; 100 ng/mL) for 7 days. The treatment significantly increased expression of p53 and p21^WAF1^ (Figure 3A), and BrdU incorporation (Figure 3B, 3C). The BrdU-positive cells became enlarged and expressed SA-β-galactosidase, a marker of senescence, along with mitochondrial elongation (Figure 3D, 3E). OXPHOS complex-IV and -V expression levels were also increased in the Doxo-induced senescence compared to that in young and old cells (Figure 3F). In contrast to the lower expression of c-fos, expression levels of p21^WAF1^ and mitochondrial genes-mtND4, COX1 and ATPase 8-were higher in the Doxo-treated young cells (Figure 3G, *p < 0.01* *vs.* young). Indeed, expression levels of the mitochondrial-OXPHOS subunit genes, such as NDUFA7 and COX17, were significantly increased in the senescent fs-HDF cells (Supplementary Figure 6). The PGC-1α, NRF1, and TFAM mRNA levels were also increased in the Doxo- *versus* vehicle-treated cells (Figure 3H, *p < 0.01*). The above phenotypes were accompanied by increased activation of PKCζ, LKB1, and AMPK compared to control cells (Figure 3I). To confirm whether PKCζ regulates the LKB1-AMPK signal axis during senescence, Doxo-treated senescent cells were transfected with siRNA-PKCζ and then analyzed by RT-qPCR. PKCζ knockdown significantly reduced the expression of TFAM and its target genes -ND4, COX1, and ATPase 8- compared to the siControl-transfected cells (Figure 3J, *p < 0.01*). In addition, the BrdU incorporation induced by Doxo treatment was also reversed by PKCζ knockdown (Figure 3K, 3L). The data indicate that mitochondrial biogenesis and nucleoid remodeling were activated in senescent fs-HDF cells *via* the PKCζ-LKB1-AMPK pathway.

**Figure 3:**
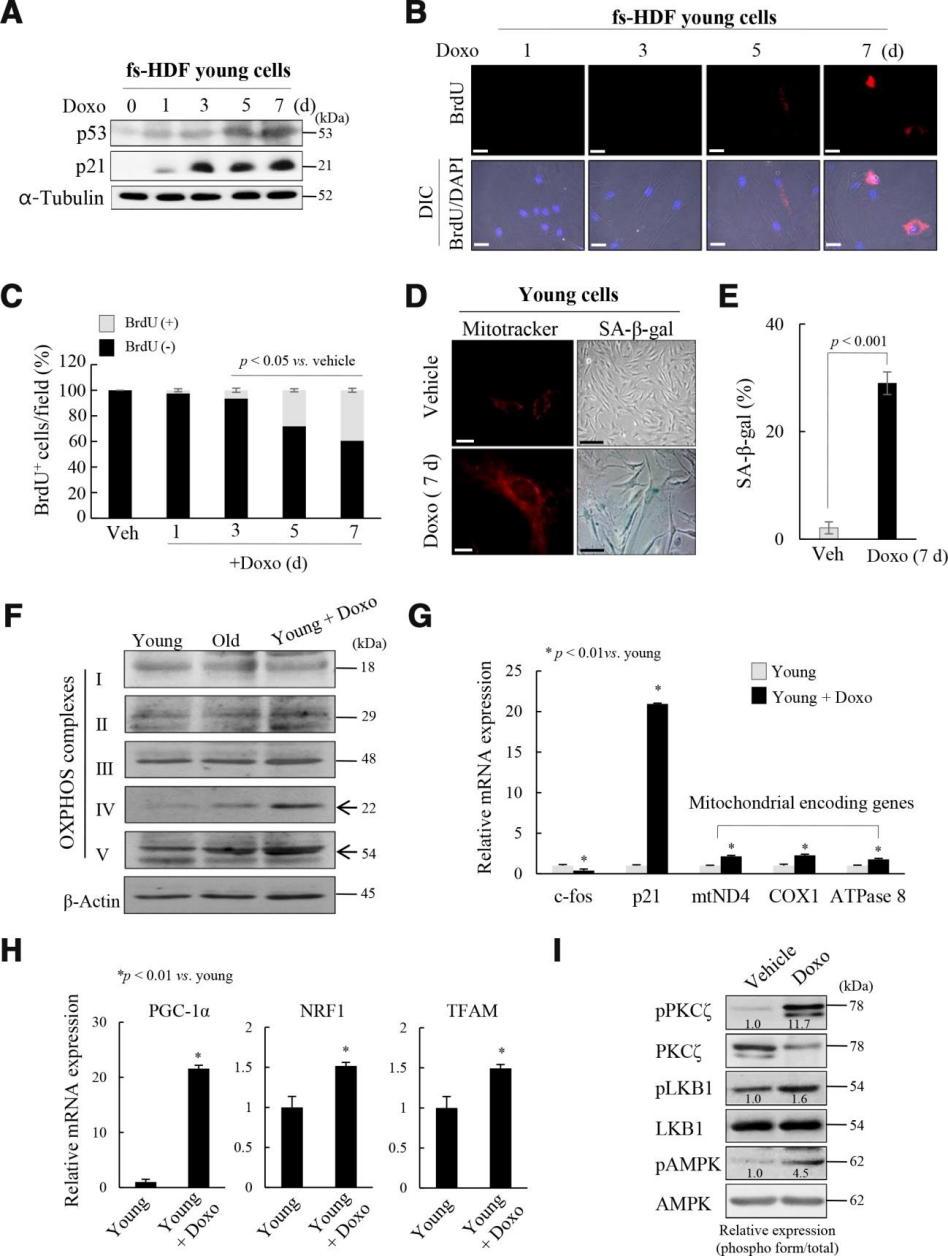
**Mitochondria are activated in senescent fs-HDF cells.** To confirm the role of PKCζ in the regulation of mitochondrial nucleoid remodeling, fs-HDF young cells were treated with doxorubicin (Doxo; 100 ng/mL) for 7 days and senescence was monitored. (**A**) Immunoblot analysis shows induction of p53 and p21^WAF1^ expression. (**B**) Doxo treatment induced BrdU incorporation at 5 days, the magnitude of which was greater at 7 days. BrdU-positive cells were examined in the dark, and DAPI fluorescence was observed under a differential interference contrast (DIC) fluorescence microscope (Carl Zeiss MicroImaging GmbH). Scale bars, 20 μm. (**C**) BrdU incorporation in mitochondria was quantified. Fluorescence microscope images were captured and counted at least 180 cells using ImageJ software (n=8 images/group). (**D**) Young fs-HDF cells and Doxo-treated (7 days) senescent cells were stained with MitoTracker or SA-β-galactosidase. Scale bars, 10 μm (white bar) or 50 μm (black bar). (**E**) Percent of SA-β-galactosidase (+) cells. Over 250 cells in 5 fields were counted. (**F**) fs-HDF young, old, and Doxo-treated young cells were subjected to immunoblot analysis, and then protein expressions of mitochondrial OXPHOS subunits were examined. (**G**) RT-qPCR analysis of the expression of complex-I, -IV, and -V subunits in young and Doxo-induced senescent cells. c-fos and p21^WAF1^ were used as the positive controls for proliferation and cell-cycle arrest, respectively. (**H**) RT-qPCR analysis of PGC-1α, NRF1 and TFAM expression in Doxo-induced senescent cells. (**I**) Immunoblot analysis of the expression of PKCζ, LKB1, and AMPK in Doxo-treated young cells and control cells. Band intensity was quantified using ImageJ software and normalized to amount of each protein. Effect of Doxo treatment was presented by the relative intensity of phosphorylation based on that of the vehicle treatment.

**Figure 3:**
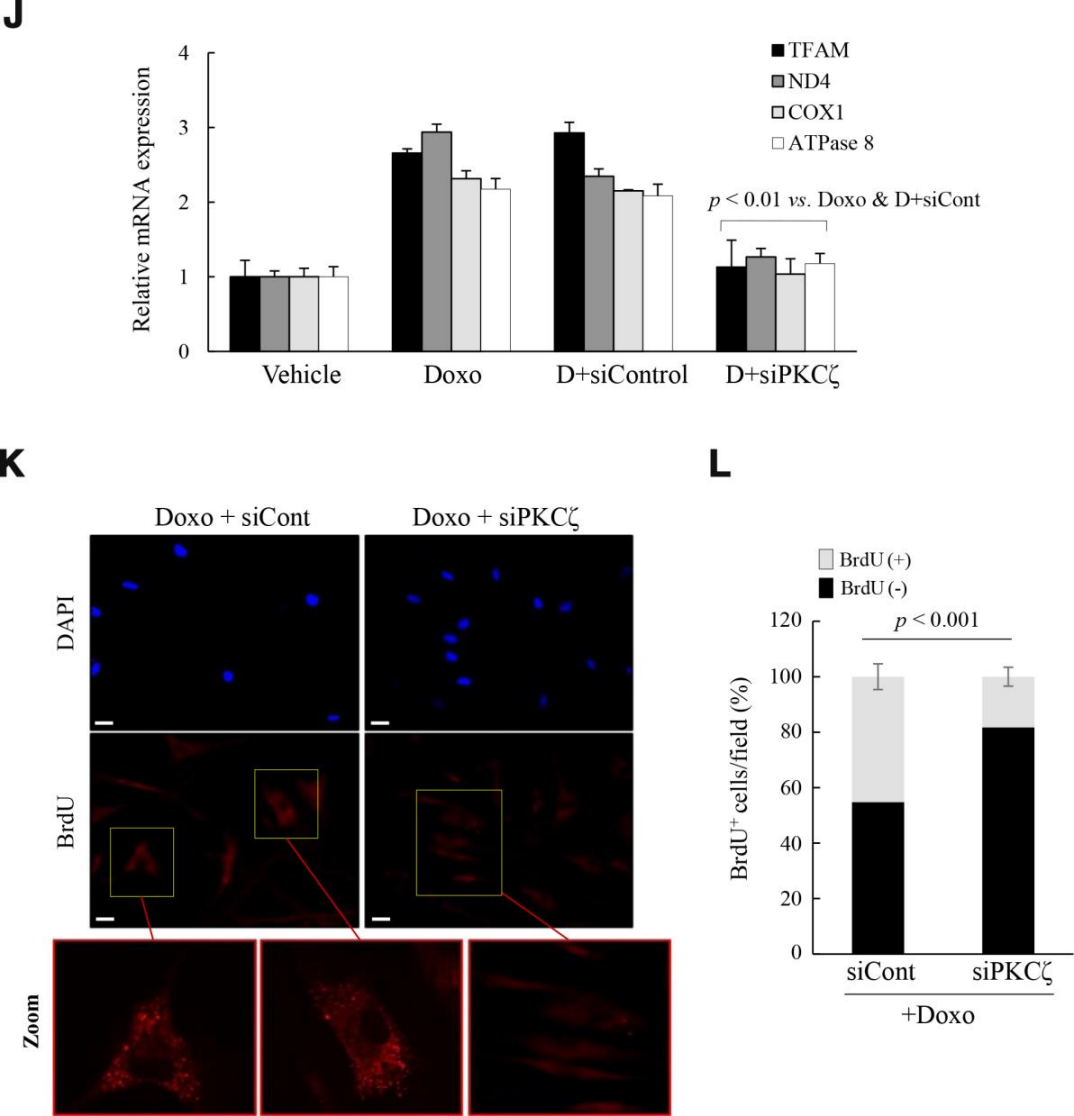
**Mitochondria are activated in senescent fs-HDF cells.** To confirm the role of PKCζ in the regulation of mitochondrial nucleoid remodeling, fs-HDF young cells were treated with doxorubicin (Doxo; 100 ng/mL) for 7 days and senescence was monitored. (**J**) Doxo-treated young cells were transfected with siControl or siPKCζ for 48 h and subjected to RT-qPCR analysis. Note the effect of PKCζ knockdown on mitochondrial biogenesis. (**K**) Fluorescence microscopy of mitochondrial nucleoid remodeling in Doxo-induced senescent cells. BrdU incorporation (red) in enlarged mitochondria was lost in the siPKCζ-transfected cells. Scale bars, 20 μm. (**L**) BrdU incorporation in mitochondria was quantified. Fluorescence microscope images were captured and counted at least 200 cells using ImageJ software (n=10 images/group). Data are means ± SD of three independent experiments per group. One-way ANOVA followed by Tukey HSD *post hoc* test.

### Activation of p21^WAF1^ regulates mitochondrial reprogramming in senescent WI-38 cells

**Figure 4:**
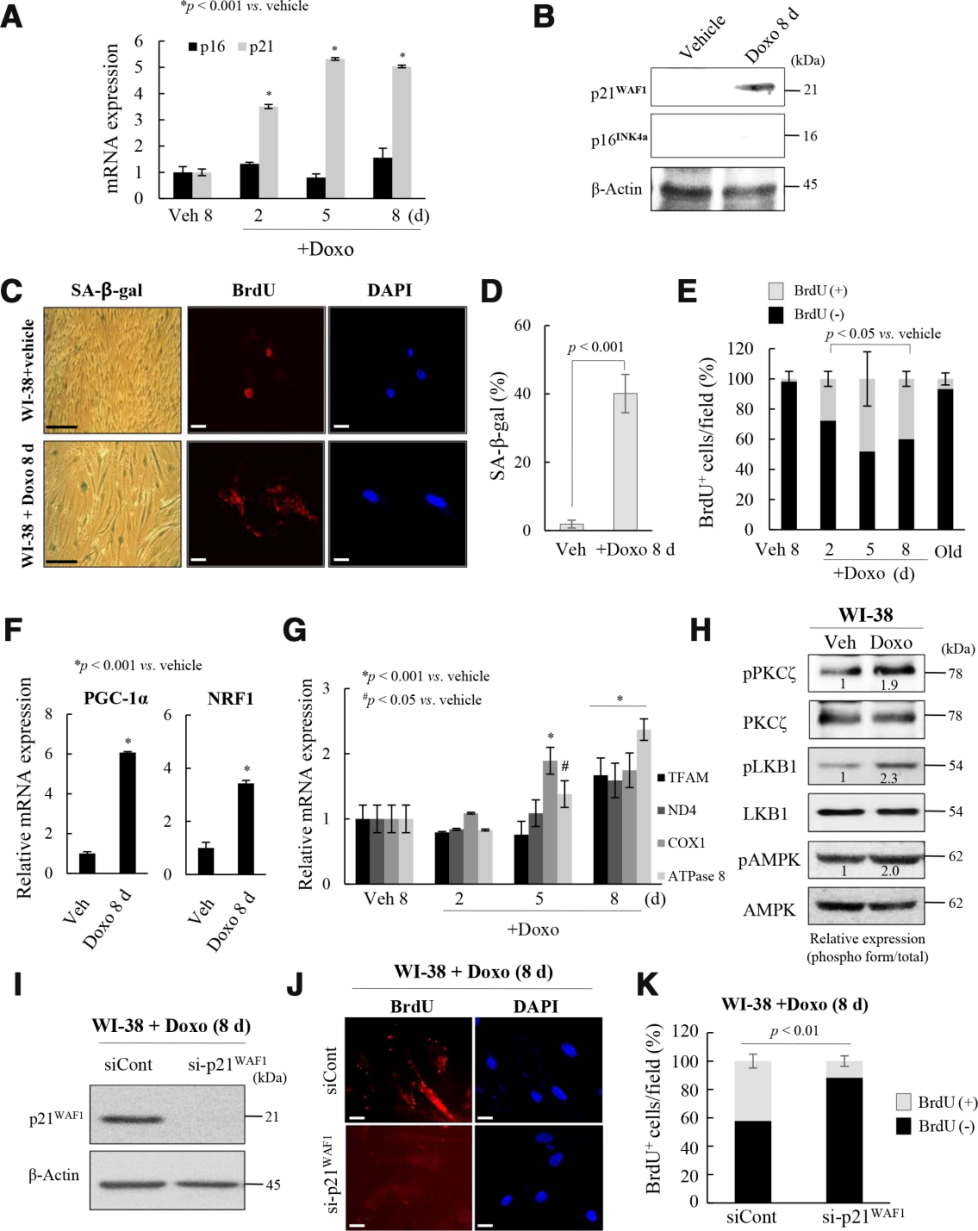
**Activation of p21^WAF1^regulates mitochondrial reprogramming in senescent WI-38 cells.** (**A**) Young WI-38 cells were treated with DOXO (100 ng/mL) for 24 h and maintained for 8 days; the expression of p21^WAF1^ and p16^INK4a^ was assayed by RT-PCR at the indicated times. (**B**) Immunoblot analysis. Note induction of p21^WAF1^, but not p16^INJ4a^, expression in young cells after Doxo treatment. (**C**) SA-β-galactosidase expression and BrdU incorporation in mitochondria in young cells after Doxo treatment for 8 days. Nuclei were stained with DAPI. Scale bars, 100 μm (black bar) or 20 μm (white bar). (**D**) BrdU incorporation in mitochondria was quantified. More than 300 cells were counted in 5 fields. (**E**) Cells with BrdU (+) mitochondria were counted under a confocal microscope using ImageJ software (n = 20 images/group). (**F**) RT-qPCR analysis of PGC-1α and NRF1 expression in Doxo-treated WI-38 young cells. (**G**) The expression of TFAM and the complex-I, -IV, and -V subunits in Doxo-induced senescent WI-38 cells was measured by RT-qPCR. (**H**) Immunoblot analysis reveals activation of PKCζ, LKB1 and AMPK after Doxo treatment. Band intensity was quantified using ImageJ software. (**I**) Doxo treated WI-38 cells were transfected with siRNAs-p21^WAF1^ and subjected to immunoblot analysis. (**J**) Immunocytochemistry showing the loss of BrdU incorporation in mitochondria by knockdown of p21^WAF1^ expression in Doxo-treated cells. Nuclei were stained with DAPI. Scale bars, 20 μm. (**K**) BrdU incorporation in mitochondria was quantified. Confocal microscope images were captured and counted at least 200 cells using ImageJ software (n=10 images/group). Data are means ± SD of three independent experiments per group.

To explore the role of the p53-p21^WAF1^ and p16^INK4a^-pRB pathways in the regulation of mitochondrial reprogramming in senescent fs-HDF cells, young WI-38 cells were treated with a low dose of Doxo for 24 h, and maintained for 8 days. The treatment significantly increased the mRNA and protein levels of p21^WAF1^, but not p16^INK4a^, in WI-38 cells (Figure 4A, 4B), accompanied by BrdU incorporation in mitochondria and SA-β-galactosidase (Figure 4C, 4D). In contrast, vehicle treatment induced BrdU incorporation in the nuclei of WI-38 cells. The number of BrdU (+) mitochondria was significantly increased in Doxo-induced senescent WI-38 cells, but not in the replicative old cells (Figure 4E). Moreover, PGC-1α and NRF1 expression was significantly induced in the Doxo-treated cells (Figure 4F, *p < 0.001*) as was that of TFAM, ND4, COX1, and ATPase 8 (complex-I, -IV, and -V, respectively, Figure 4G, *p < 0.001*). PKCζ, LKB1, and AMPK were all activated in Doxo-induced senescence of WI-38 cells (Figure 4H), and the knockdown of p21^WAF1^ expression significantly reduced BrdU incorporation into the Doxo-induced senescent cells (Figure 4I–4K, *p* < *0.01*). These data imply that induction of p21^WAF1^, but not 16^INK4a^, expression regulates mitochondrial nucleoid remodeling and biogenesis in senescent human fibroblasts.

### Silencing of p16^INK4a^ but not p21^WAF1^ induces mitochondrial remodeling in senescent human fibroblasts

**Figure 5:**
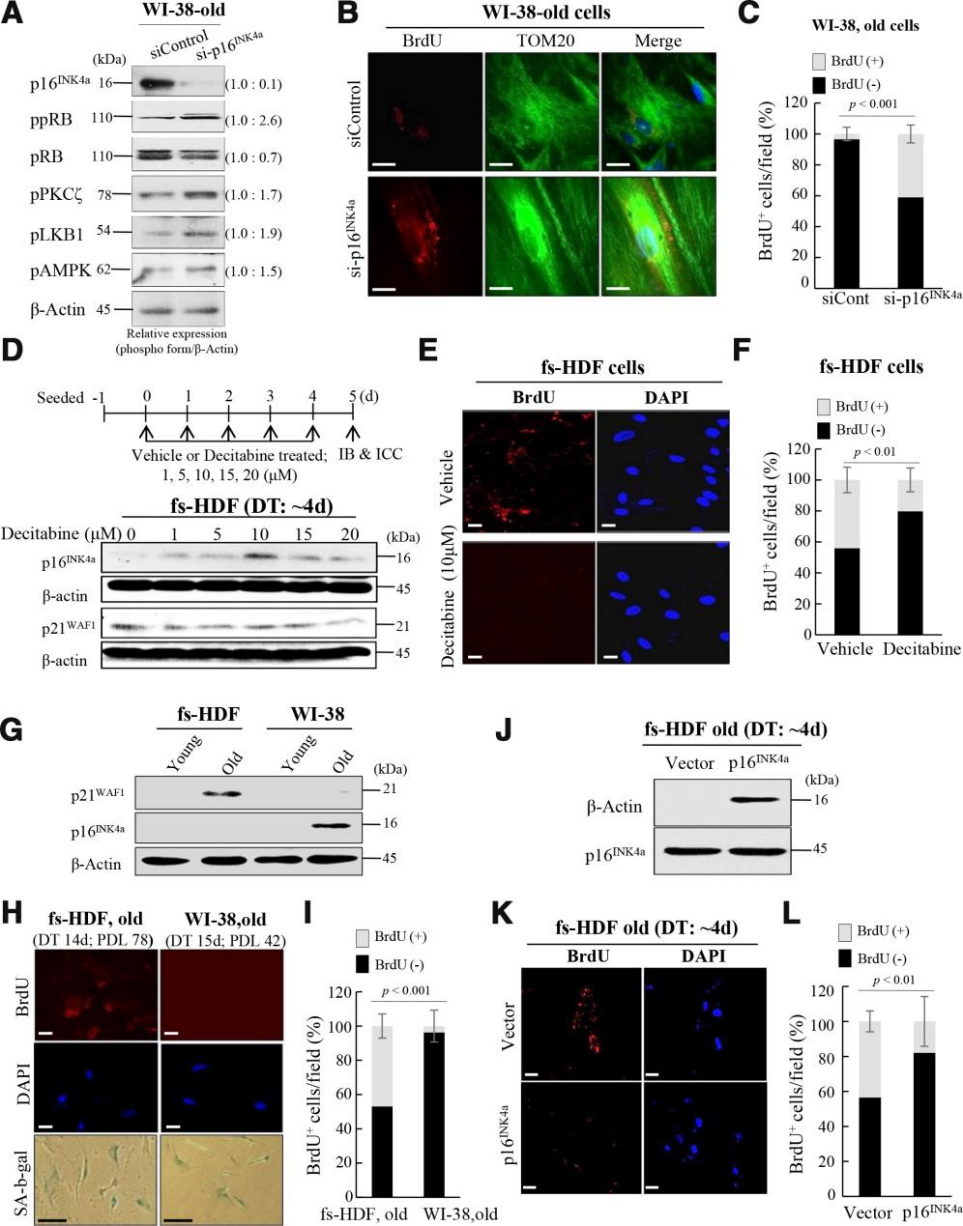
**Silencing of p16^INK4a^ or induction of p21^WAF1^ regulates mitochondrial remodeling in human fibroblasts.** (**A**) Immunoblot analysis after knockdown of p16^INK4a^ by transfection of old WI-38 cells with siRNA-p16^INK4a^. Note increase of pRB phosphorylation by knockdown of p16^INK4a^ expression along with activation of the PKCζ-LKB1-AMPK signals. Band intensities were calculated by ImageJ software and normalized to that of β-Actin. (**B**) Knockdown of p16^INK4a^ expression induced mitochondrial nucleoid formation in old WI-38 cells. Note anti-BrdU (red) and anti-Tom20 (green) expression in the siRNA-p16^INK4a^-transfected old WI-38 cells. Nuclei were stained with DAPI (blue). Scale bars, 10 μm. (**C**) Mitochondrial incorporation of BrdU was quantified. Confocal microscope images were captured and counted at least 120 cells using ImageJ software (n=6 images/group). (**D**) Induction of p16^INK4a^ expression by repeated treatment of mid-old fs-HDF cells with decitabine (1–20 μM). The expression level of p16^INK4a^ was higher after 5 days of 10 μM treatment, whereas that of p21^WAF1^ was constant. (**E**) Loss of mitochondrial nucleoid formation in fs-HDF mid-old cells treated with decitabine (10 μM) for 5 days. Scale bars, 20 μm. (**F**) Quantification of BrdU (+, -) mitochondria. Confocal microscope images were captured and counted at least 180 cells using ImageJ software (n = 8 images/group). (**G**) Significant induction of p21^WAF1^ expression in old fs-HDF cells, in contrast to p16^INK4a^ expression in old WI-38 cells. (**H**) Comparison of old fs-HDF and old WI-38 cells. Note that 78 and 42 PDLs differed between the two cell types, respectively, despite the similar doubling times (DTs). Nucleoid remodeling was observed in the old fs-HDF cells, but not in the old WI-38 cells. Scale bars, 20 μm (white bar) or 100 μm (black bar). (**I**) Mitochondrial incorporation of BrdU was quantified. Confocal microscope images were captured and counted at least 180 cells using ImageJ software (n=9 images/group). (**J**) Mid-old fs-HDF cells were transfected with pCMV-p16^INK4^ and subjected to immunoblot analysis. (**K**) Loss of mitochondrial nucleoid formation in mid-old fs-HDF cells after forced expression of p16^INK4^ gene for 6 days. Scale bars, 20 μm. (**L**) Mitochondrial incorporation of BrdU was quantified. Confocal microscope images were captured and counted at least 150 cells using ImageJ software (n=7 images/group).

To clarify the effects of the p53-p21^WAF1^ and 16^INK4a^-pRB pathways on mitochondrial remodeling, expression of p16^INK4a^ was reduced by transfection of WI-38 old cells (DT: 15 days) with siRNA-p16^INK4a^ (by ~ 90%), which was also evaluated by phosphorylation of pRB, and the PKCζ-LKB1-AMPK axis was activated by the knockdown (Figure 5A). When mitochondrial nucleoid remodeling was observed by confocal microscopy, BrdU incorporation was induced in mitochondria of the p16^INK4a^ reduced cells (Figure 5B, 5C), indicating that suppression of p16^INK4a^ expression stimulated mitochondrial nucleoid formation, even in WI-38 old cells. To investigate further the role of p16^INK4a^ in regulation of mitochondrial nucleoid remodeling, expression of p16^INK4a^ was induced by treating senescent fs-HDF cells (DT: 4 days) with decitabine, a demethylation agent. The treatment induced p16^INK4a^ expression (Figure 5D), and prevented BrdU incorporation in the senescent cells (Figure 5E, 5F). Basal level p21^WAF1^ expression was prominent in old fs-HDF cells, whereas old WI-38 cells highly expressed p16^INK4a^ (Figure 5G). The two cell lines exhibit significantly different rates of senescence development; the numbers of population doublings (PDLs) with a DT of > 14 days were 78 and 42 in fs-HDF and WI-38 cells, respectively (Figure 5H), and the mitochondria of old WI-38 cells failed to incorporate BrdU (Figure 5I). On the other hand, overexpression of p16^INK4a^ could reduce BrdU incorporation in senescent fs-HDF cells (Figure 5J–5L), implying that expression of p16^INK4a^ suppresses mitochondrial nucleoid remodeling.

**Figure 6:**
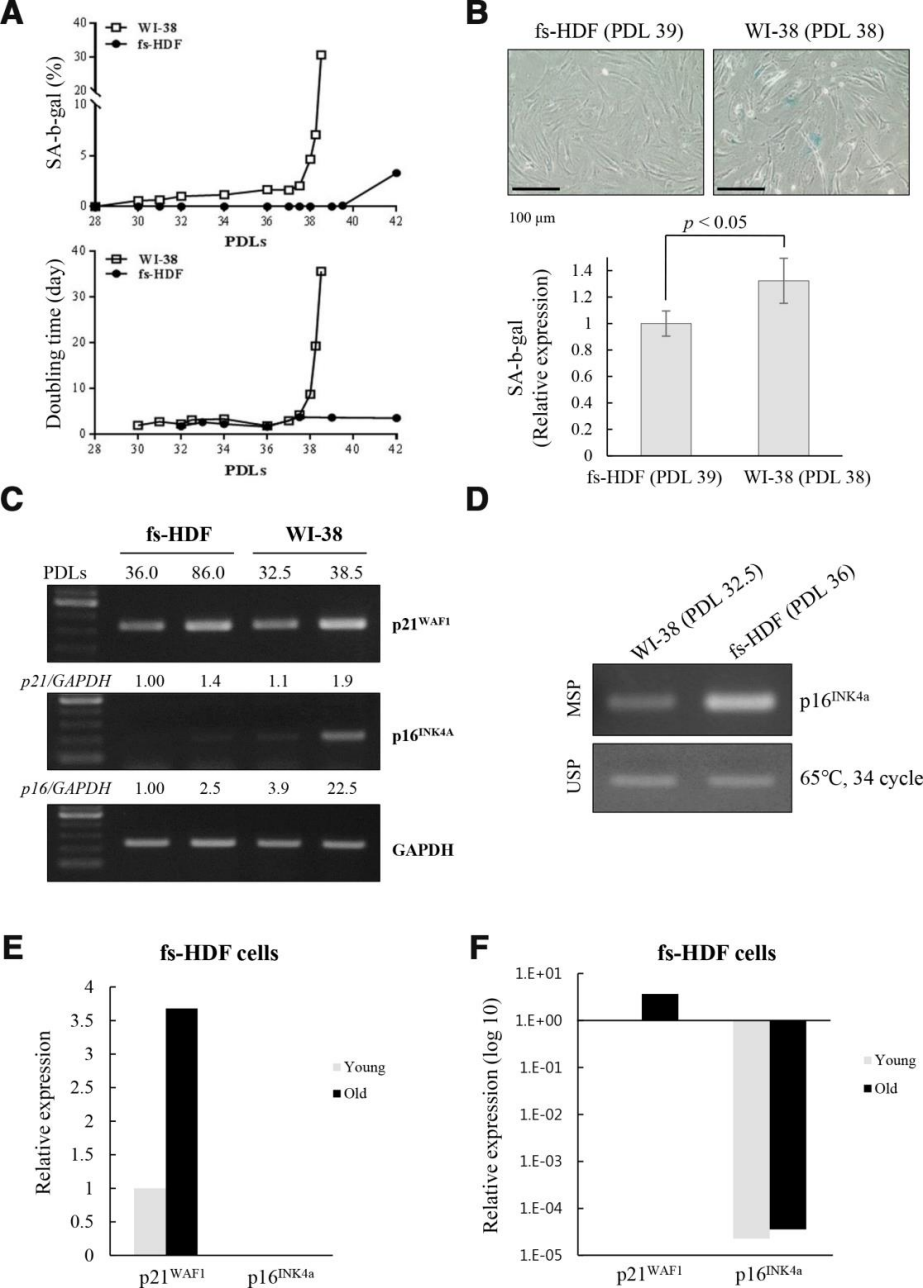
**Replicative senescence in fs-HDF cells with p16^INK4a^ silenced is delayed relative to WI-38 cells expressing elevated p16^INK4a^.** (**A**) Earlier induction of SA-β-galactosidase in WI-38 cells compared to fs-HDF cells accompanied by significant differences in their doubling times and number of population doublings (PDLs). (**B**) WI-38 cells are positive for SA-β-galactosidase at 38 PDL, but fs-HDF cells at 39 PDL are not. (**C**) RT-qPCR analysis showing no induction of p16^INK4a^ expression in fs-HDF cells at 86 PDL in contrast to marked induction in WI-38 cells at 38.5 PDL. (**D**) Methylation-specific PCR (MSP) and unmethylation-specific PCR (USP) analysis. The methylation status of the p16^INK4a^ gene differed slightly between WI-38 and fs-HDF cells. (**E**) Relative expression of p21^WAF1^ and p16^INK4a^ genes in old and young fs-HDF cells, as seen in RNA sequencing analysis. p16^INK4a^ expression was almost absent in fs-HDF cells. (**F**) The data presented in (**E**) was log10 transformed. Note the absence of p16^INK4a^ expression in young and old fs-HDF cells, in contrast to clear induction of p21^WAF1^ expression in the old cells.

When analyzed the different rates of senescence in the fs-HDF and WI-38 cells, the increase of DT and SA-β-galactosidase expression was markedly delayed in fs-HDF compared to WI-38 cells; the DT was dramatically increased in WI-38 cells after 38 PDLs along with marked expression of SA-β-galactosidase, whereas fs-HDF cells actively proliferated without expressing SA-β-galactosidase until 42 PDLs (Figure 6A, 6B). In addition, p16^INK4a^ expression was markedly increased (22.5-fold) in WI-38 cells at 38.5 PDLs than 32.5 PDLs; however, expression of p21^WAF1^ and p16^INK4a^ was slightly increased in fs-HDF cells at 86 PDLs *versus* 36 PDLs (Figure 6C). The degree of DNA methylation in the promoter region of p16^INK4a^ differed slightly between the WI-38 and fs-HDF cells (Figure 6D). To examine further the expression patterns of p21^WAF1^ and p16^INK4a^ in fs-HDF cells, RNA-sequencing analysis was performed. The expression of p21^WAF1^ was increased in the old cells compared to the young cells; however, p16^INK4a^ expression was out of detection in young and old fs-HDF cells (Figure 6E). When the data were log10 transformed, p16^INK4a^ expression was absent in young and old fs-HDF cells, despite the higher p21^WAF1^ expression in the old cells compared to the young cells (Figure 6F). In summary, fs-HDF cells failed to induce expression of p16^INK4a^ that regulates nucleoid remodeling and senescence. Here we suggest that activation of the p53-p21^WAF1^ pathway along with silencing of p16^INK4a^ might influence senescence development in human fibroblasts *via* inducing mitochondrial remodeling.

## DISCUSSION

**Figure 7:**
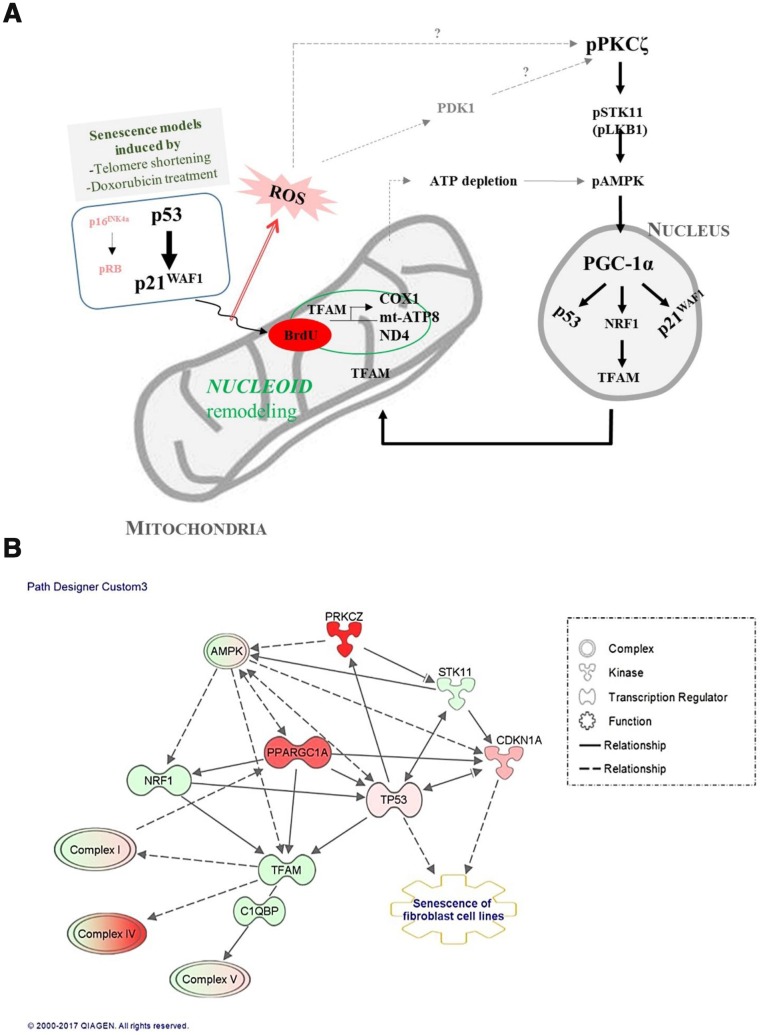
**Signal transduction pathways regulate mitochondrial nucleoid remodeling and biogenesis in senescent human fibroblasts.** (**A**) Activation of p53-p21^WAF1^ or silencing of p16^INK4a^-pRB induces mitochondrial nucleoid remodeling and biogenesis *via* the PKCζ-LKB1(STK11)-AMPK-PGC-1α-NRF1-TFAM pathway in senescent human fibroblasts. Induction of p21^WAF1^ and silencing of p16^INK4a^, together with reactive oxygen species (ROS) accumulation and PDK1, significantly activated PKCζ, which might explain the delayed senescence in fs-HDF compared to WI-38 cells. (**B**) Ingenuity pathway analysis (IPA) using RNA-sequencing data. Red and pink indicate increased expression; green shows decreased expression. Arrow indicates signal activation; solid line for direct interaction, dotted line shows possible interaction. Note significant activation of PKCζ and PGC-1α (PPARGC1A), and mild activation of AMPK. Increased expression levels of TP53 and CDKN1A (p21^WAF1^) were followed by senescence of human fibroblasts and biogenesis of the mitochondrial complex-I, -IV, and -V subunits.

Mitochondrial activity and biogenesis are significantly reduced *in vitro* in senescent cells, and *in vivo* during aging [27, 28], and mitochondrial biogenesis and respiratory capacity support increased metabolic activity in cancer cells and promote cell motility by inducing ROS generation [29–31]. We report here that the signaling pathway that regulates mitochondrial biogenesis and mitochondrial nucleoid remodeling was activated in human fibroblasts undergoing senescence *via* the p53-p21^WAF1^ axis and PKCζ activation, when the expression of p16^INK4a^ is silent (Figure 7A). Although the number of dysfunctional mitochondria was more in senescent than in young cells, the old fs-HDF cells exhibited greater mitochondrial biogenesis, as indicated by increased TFAM and PGC-1α (PPARGC1A) expression (Figures 2A and 7B). TFAM is a major component of the mammalian nucleoid, and is involved in the maintenance of mtDNA in mammals and other vertebrates [17]. The high content of mtDNA and TFAM activities in old cells support BrdU incorporation and TFAM colocalization in mitochondrial nucleoids (Figure 1A, Supplementary Figure 1). Active transcription of the mitochondrial OXPHOS complex I and IV subunits (Figure 1C, 1D) also supports the remodeling of mitochondrial nucleoids in old fs-HDF cells, to compensate for partial uncoupling of oxidative phosphorylation and insufficient ATP production despite the increased OCR [10]. However, we cannot exclude the possibility that the increased BrdU incorporation and DNA contents in the mitochondria of old cells are due to mitochondrial fusion or a nascent transcript in mitochondrial RNA granules [32]. We assume that the significantly higher PGC-1α expression (Figure 1E and Supplementary Figure 3) compensates for the senescent phenotypes of old mitochondria, which might delay senescence in fs-HDF cells compared to WI-38 fibroblasts. In contrast to the active mitochondrial biogenesis and mitochondrial nucleoid remodeling, the old fs-HDF cells exhibited senescence phenotypes such as mitochondrial elongation, an elevated OPA1 level, mitochondrial depolarization, ROS accumulation, and a low ATP level (Supplementary Figure 4).

PKC activation is regulated by either release of the N-terminus and pseudosubstrate from substrate-binding cavity or phosphorylation of the kinase domain [33]. Based on our previous report, PKCζ expression is abundant in fs-HDF cells, and activated in senescent fs-HDF cells [23, 34]. Accumulated ROS in senescent cells inactivate MKP-3 (tyrosine phosphatase) and PP1/2A (serine/threonine phosphatase) *via* oxidation of Cys residues and metal ion(s) [35]. Activation of PKCζ is mainly regulated by phosphatidyl inositols (PIs) [36], arachidonic acid and ceramide [37]. PI-3,4,5-trisphosphate is a regulator of aPKCs by direct binding to Akt and phosphoinositide-dependent kinase (PDK1) that can phosphorylate Thr-410 in the activation loop of PKCζ [38, 39]. PDK1 activity may be regulated by senescence-induced oxidative stress by tyrosine phosphorylation [40]. Based on the above notion, we assumed that activation of PDK1 by ROS might stimulate PKCζ in senescent cells.

The free-radical theory of aging is supported by the accumulation of dysfunctional mitochondria with aging. However, the levels regulating mitochondrial biogenesis reportedly increase in cellular senescence and aging; upregulation of TFAM and NRF1 in the age-related increase in mtDNA content in human lung fibroblasts, osteosarcoma 143B TK and skeletal muscle [29, 41], and increased expression of NRF2α, PGC-1α, PGC-1β, and TFAM in *ras*-induced senescence [42]. In oncogene-induced senescence, mitochondrial metabolism is upregulated along with the metabolic demand for cytokine production, such as IL-6, [43]. Although there are other possibilities, we focused on the role of PKCζ in the regulation of AMPK activity through phosphorylation of Ser^428^ by LKB1 in senescence [21]. As compared to young cells, PKCζ-LKB1 and AMPK activation was significantly higher in old cells, and knockdown or inhibition of PKCζ expression was accompanied by reduced expression of TFAM and its target genes, as well as BrdU incorporation in mitochondria of old cells. The data support a role of PKCζ in regulating mitochondrial activity (Figure 2 and Supplementary Figure 5). The changes observed in replicative senescence could be reproduced in Doxo-induced senescence of young fs-HDF cells (Figure 3). Therefore, active mitochondrial nucleoid remodeling and biogenesis are induced *via* the PKCζ -LKB1-AMPK pathway in fs-HDF old cells.

When the senescence program of fs-HDF and WI-38 cells were examined, the rate of senescence development was vary different (Figure 6); Induction of replicative senescence was markedly delayed in fs-HDF cells compared to WI-38 cells, as evaluated by SA-β-galactosidase expression and doubling times based on the number of PDLs (Figure 6A, 6B). WI-38 cells transmitted more senescence signals *via* p16^INK4a^ activation compared to fs-HDF cells (Figure 6C). There was no expression of p16^INK4a^ in fs-HDF cells, despite the mild induction of p21^WAF1^ expression in replicative senescence, as measured by RNA sequencing (RNA-Seq; Figure 6E, 6F). In contrast to the significant induction of BrdU incorporation in the mitochondria of old fs-HDF cells, the finding was not detected in old WI-38 cells unless the p53-p21^WAF1^ pathway was activated by Doxo (Figure 4 and Supplementary Figure 7), indicating that the signals regulating mitochondrial remodeling in senescence require activation of the p53-p21^WAF1^ axis and the PKCζ-LKB1-AMPK pathway if p16^INK4a^ is silenced. The activation of the PKCζ-LKB1-AMPK-p21^WAF1^ pathway is supported by the IPA of DEGs identified by RNA-Seq in fs-HDF cells. Silencing of p16^INK4a^, a prerequisite for mitochondrial BrdU incorporation, was evident in fs-HDF cells (Figure 5). The Ink4/Arf locus is completely silenced in embryonic stem cells and can be reactivated by differentiation [44]; thus, silencing is not the result of a selective process but intrinsic to the differentiation of fs-HDF cells. p16^INK4a^ silencing was required for mitochondrial nucleoid remodeling in fs-HDF and WI-38 cells, and the regulation of p16^INK4^ expression by siRNAs and decitabine in WI-38 and fs-HDF cells, respectively, changed induction of the nucleoid remodeling. Indeed, p16^INK4a^ regulates cell cycle *via* the CDK4/Rb-dependent pathway [45], and modulates mitochondrial dynamics and cell motility in mouse fibroblasts and human melanoma cells [46]. We report here the role of p16^INK4a^ as a regulator of mitochondrial nucleoid remodeling and mitochondrial biogenesis during induction of senescence *via* the p53-p21^WAF1^ pathway. p16^INK4a^ silencing in senescent cells may compensate for the functional decline in mitochondria through the PKCζ-LKB1-AMPK pathway, leading to p53-p21^WAF1^ activation. Therefore, the delayed induction of senescence in fs-HDF compared to WI-38 cells might have been due to activation of the p53-p21^WAF1^ axis under silencing of p16^INK4a^. The notion is accordant with a previous report that DNA damage *via* p53 induction is required for regulation of mitochondrial nucleoid remodeling [15]. Present study provides insight into the aging-associated changes in mitochondria that regulate the degree and rate of senescence induction. In summary, the PKCζ-LKB1-AMPK signals regulate nucleoid remodeling *via* activation of the p53-p21^WAF1^ pathway, which is indispensable for senescence induction under p16^INK4a^ silencing, and stimulate PGC-1α-NRF1-TFAM expression for mitochondrial biogenesis.

## MATERIALS AND METHODS

### Preparation and culture of fs-HDFs

The fs-HDFs were isolated as described previously [47], and subjected to short tandem repeat (STR) profiling at Ajou University Hospital. Primary cultures of fs-HDF and established WI-38 lung fibroblasts were maintained in Dulbecco’s modified Eagle’s medium supplemented with 10% fetal bovine serum (Invitrogen/Gibco, Grand Island, NY, USA) and the numbers of PDLs and their DTs were calculated as described previously [23]. The DT of young cells was about 26 h, and those of mid-old and old cells were 4–10 and > 14 days, respectively. Cells were incubated at 37°C with 5% CO_2_ in air.

### STR profiling

Genomic DNAs isolated from cells were mixed with 4 μL of AmpFlSTR PCR reaction mix, 2 μL of Identifiler primers and 0.2 μL of Amplitaq Gold DNA polymerase (Thermo Fisher Scientific, Waltham, MA, USA) in a 10-μL reaction volume. The PCR conditions were: initial amplification at 94°C for 11 min, followed by 28 cycles at 94°C for 1 min, 59°C for 1 min, and 72°C for 1 min, with a final amplification at 60°C for 1 h. The PCR products were analyzed on a Gene Sequencer (ABI 3500XL; Applied Biosystems, Foster City, CA, USA) with GeneScan software (Applied Biosystems).

### Doxorubicin treatment

Young fs-HDF or WI-38 cells were seeded at 8 × 10^4^ on a six-well plate with or without a coverslip, and exposed to Doxo (100 ng/mL) for 24 h. The cells were maintained in complete medium, which was changed every 3 days. Doxo-induced cellular senescence was monitored by assaying the expression of p53, p21^WAF1^, and p16^INK4a^ at the indicated times.

### Senescence-associated-β-gal assay

Cells were washed twice in phosphate-buffered saline (PBS), fixed for 5 min in 4% formaldehyde, and incubated at 37°C overnight with 1.0 mg X-Gal/mL dimethylformamide, 40 mM citric acid/sodium phosphate (pH 6.0), 5 mM potassium ferrocyanide, 5 mM potassium ferricyanide, 150 mM NaCl and 2 mM MgCl_2_. Staining was evident at 2–4 h and peaked at 12–16 h.

### Immunoblotting

Cells were solubilized in radioimmunoprecipitation assay buffer (RIPA) buffer (50 mM Tris/HCl [pH 7.5], 150 mM NaCl, 1.0% Nonidet P40, 0.1% sodium dodecyl sulfate (SDS), 0.5% deoxycholic acid, 1.0 μg/mL leupeptin, 100 μg/mL phenylmethylsulfonyl fluoride, 1.0 mM Na_3_VO_4_, and 1.0 mM NaF) and cleared by centrifugation at 12,000 g for 10 min at 4°C. Next, 40 μg of the lysate/lane was resolved by 10–15% SDS-polyacrylamide gel electrophoresis (SDS-PAGE) in 25 mM Tris/glycine buffer. The protein bands were transferred to a polyvinylidene fluoride membrane, treated with 5% non-fat skim milk in PBS containing 0.05% Tween 20 (PBST) for 1 h, and incubated with the indicated antibodies at 4°C overnight. The membrane was washed three times with PBST and incubated with the horseradish peroxidase-conjugated secondary antibodies for 1 h. An enhanced chemiluminescence (Amersham Biosciences, Little Chalfont, UK) kit was used to visualize the protein bands.

### PKCζ kinase assay

Immunoprecipitation (IP) was performed with cell lysates (~ 1.0 mg of protein) using the standard method in RIPA buffer without 0.1% SDS. Whole-cell lysates were pre-cleared using protein G-agarose beads (Invitrogen) for 1 h and precipitated overnight with an anti-PKCζ antibody at 4°C. The immunoprecipitates were washed five times with IP buffer and PKCζ was eluted using IgG elution buffer (Thermo Scientific, Wilmington, DE, USA). A PKC-specific kinase assay was performed using a PKC Activity Assay Kit (Abcam, Cambridge, UK) according to the manufacturer’s instructions.

### Immunocytochemistry

Coverslip cultures were washed with PBS, fixed in 4% paraformaldehyde for 15 min, permeabilized with 0.05% Triton X-100, and incubated with 3% bovine serum albumin (BSA) in 0.05% Triton X-100 for 2 h. The primary antibody was applied at 4°C overnight, followed by the secondary antibody for 2 h. Next, the coverslips were stained with 4% 6-diamidino-2-phenylindole (DAPI; 2.0 mg/mL) for 10 min at room temperature and mounted. Expression of BrdU and TFAM was determined using monoclonal or polyclonal antibodies conjugated to Cy3 and Alexa 488, and a goat-anti-mouse IgG secondary antibody conjugated to Alexa 555 or a goat-anti-rabbit IgG. The cells were visualized under a fluorescence microscope, and images were obtained using an AxioVision imager and analyzed *in silico* (Carl Zeiss MicroImaging GmbH, Jena, Germany).

### BrdU incorporation assay

Cells treated with Doxo for 24 h were transfected with siControl or siRNAs for 48 h. The cells were labeled with 10 μM BrdU (Sigma) for 4 h, fixed in 4% paraformaldehyde, and incubated in 2 M HCl for 30 min. The pH was increased by adding 0.1 M sodium borate (pH 8.5) for 2 min. Immunocytochemistry was performed using an anti-BrdU or anti-TFAM antibody, and positive cells were visualized under a fluorescence or confocal microscope.

### Real-time PCR

Total cellular RNA was extracted using RNAiso Plus (TaKaRa, Shiga, Japan), and cDNA was synthesized from 1.0 μg of RNA using a Reverse Transcription Kit (Invitrogen, Carlsbad, CA, USA). cDNA was amplified in a CFX96 Real-Time PCR Cycler (Bio-Rad, Hercules, CA, USA) using specific primers and SYBR Green PCR Master Mix (Applied Biosystems) under the following conditions: activation at 95°C for 15 min, followed by 40 cycles of 95°C for 20 s and 60°C for 40 s. The primers are listed in Supplementary Table 1.

### Transfection with short interfering RNAs

siRNAs were purchased from Santa Cruz Biotechnology (Santa Cruz, CA, USA). Cells on coverslips were transfected with control siRNAs (sc-37007), siPKCζ (sc-44270), si-p21^WAF1^ (sc-29427) and si-p16^INK4a^ (sc-37622) using Lipofectamine RNAiMAX (Invitrogen). After 48 h, the cells were subjected to further analyses. To exclude off-set target by siRNA transfection, we used the siRNAs containing more than 3 sets of mixture.

### Plasmid transfection

pCMV-p16^INK4a^ was purchased from Addgene (Watertown, MA, USA.). HDF cells in six-well plates (5 × 10^4^/well) were transiently transfected with 2 μg of a vector or pCMV-p16^INK4a^ using Lipofectamine 3000 reagent (Thermo Fisher Scientific) and incubated for 48 h. After that the cells were analyzed by immunoblotting method.

### Electron microscopy

Young and old fs-HDF cells were post-fixed in 1% osmium tetroxide, dehydrated in 70–100% ethanol, incubated in propylene oxide, and then embedded in Embed 812 resin (Electronic Microscopic Science, Hatfield, PA, USA). Mitochondria were observed under an EM902A microscope (Carl Zeiss MicroImaging GmbH,) at 12,000-fold magnification.

### Preparation of the mitochondrial fraction

The mitochondrial fraction was isolated as described previously with a minor modification [48]. Trypsin-treated cells resuspended in ice-cold isolation buffer containing 0.1 M Tris-HCl (pH 7.4), 0.1 M EDTA, and 250 mM sucrose were homogenized using a glass homogenizer (30 strokes) and transferred to a new tube. The supernatant was collected by centrifugation at 600 g for 10 min, followed by at 7,000 g for 10 min at 4°C. The pellet was washed with 200 μL of ice-cold isolation buffer and centrifuged at 7,000 g for 10 min at 4°C. The final pellet was resuspended in 100 μL of buffer. The nuclear and mitochondrial fractions were verified by assaying lamin B1 and TOM20 (Santa Cruz Biotechnology, Santa Cruz, CA, USA) by SDS-PAGE.

### Mitochondrial DNA copy-number analysis

Total DNA was isolated using the buffer-saturated phenol, chloroform, and isoamyl alcohol extraction method. DNA was quantified using an Eo Microplate Spectrophotometer (BioTek, Winooski, VT, USA) by measuring absorbance at 260 and 280 nm. mtDNA content was determined as the ratio of mtDNA (NADH-ubiquinone oxidoreductase subunit 1; ND1) to nDNA of 18S rRNA by real-time PCR (CFX96, Bio-Rad) with SYBR Green PCR Master Mix (Applied Biosystems), DNA (5 ng) and specific primers (Supplementary Table 1).

### Measurement of ATP content

The amount of ATP was quantified using an ATP Determination Kit (Molecular Probes, Eugene, OR, USA), as described previously [34].

### Measurement of mitochondrial respiration

Old cells (1 × 10^4^/well) were seeded on XF24 plates, cultured in a CO_2_ incubator at 37°C for 24 h, and transfected with siRNAs for 48 h. After incubation with or without 10 μM oligomycin for 1 h, the medium was replaced with 500 μL of XF assay medium (25 mM glucose, 143 mM NaCl, 5.4 mM KCl, 0.8 mM MgSO_4_, 0.91 mM Na_2_HPO_4_, 2 mM glutamine, 2 mg/mL BSA, and 15 mg/L phenol red, pH 7.4), and incubated in a normal atmosphere for 1 h to remove CO_2_. Respiration was measured using an XF24 Extracellular Flux Analyzer (Seahorse Bioscience, Billerica, MA, USA) at 37°C.

### Synthesis of cDNA and preparation of RNA libraries

The quality and quantity of RNA were assessed using a NanoDrop1000 Spectrometer (Thermo Scientific) and Bioanalyzer 2100 (Agilent Technologies, Santa Clara, CA, USA). Next, a TruSeq Strand-Specific mRNA Library Preparation Kit (Illumina, San Diego, CA, USA) was used to construct Illumina-compatible libraries according to the manufacturer’s instructions. Briefly, 1.0 μg of total RNA was subjected to polyA-selected RNA extraction, RNA fragmentation, random hexamer-primed reverse transcription, and 100 nt paired-end sequencing on an Illumina HiSeq4000. The libraries were quantified by qPCR according to the manufacturer’s instructions, and qualified using an Agilent Technologies 2100 Bioanalyzer (Agilent Technologies). Finally, the products were sequenced on the HiSeq™ 2000 platform (Illumina).

### RNA sequencing

To estimate expression levels, the RNA-Seq reads were mapped to the human genome using TopHat (version 1.3.3; http://tophat.cbcb.umd.edu). The reference genome sequence (mm10) and annotation data were downloaded from the UCSC website (http://genome.ucsc.edu). The transcript counts were calculated, and the relative transcript abundances were measured in units of fragments per kilobase of exon per million (FPKM) fragments mapped using Cufflinks software (version 1.2.1; http://cole-trapnell-lab.github.io/cufflinks/). Functionally important genes were validated by qPCR.

### Ingenuity pathway analysis

Network interactions among genes, functions, and signaling pathways that regulate senescence phenotypes in fs-HDF fibroblasts were constructed using Ingenuity Pathway Analysis software, Version 21901358 (IPA; Ingenuity systems, Redwood City, CA, USA) by uploading the DEG data. Genes and bio-functions are represented as nodes, and biological interactions as lines. Upregulation is shown as red and downregulation as green. The color intensity of the node indicates the degree of expression.

### Statistical analysis

Data are means ± standard deviation (SD) of at least three independent experiments. The quantitative data were analyzed by Student’s *t*-test (two groups) or one-way analysis of variance (ANOVA) among multiple groups, followed by Tukey honestly significant difference (HSD) *post hoc* tests using SPSS software, Version 22.0 (SPSS Inc., Chicago, IL, USA). A *p*-value < 0.05 was considered indicative of significance.

## Supplementary Materials

Supplementary Methods

Supplementary Figures

Supplementary Table 1
