# Supplementary material for: Mitochondrial nucleoid remodeling and biogenesis are regulated by the p53-p21WAF1-PKCζ pathway in p16INK4a-silenced cells
Source: Aging (Albany NY). 2020 Apr 24;12(8):6700–32. doi: 10.18632/aging.103029 (PMC7202532; doi:10.18632/aging.103029)
Supplement: Supplementary Methods [file aging-12-103029-s001..pdf]

SUPPLEMENTARY METHODS

Detection of ROS level

Young and old cells were incubated with 10 μM DCFDA for 30 min at 37 °C and then washed with PBS for 3 times. Cells were trypsinized before re-suspended in PBS, and DCF fluorescence was measured by flow cytometry (BD Biosciences).

Mitochondrial membrane potential changes

Young and old cells were stained with 5, 5′, 6, 6′-tetrachloro-1, 1′, 3, 3′-tetraethylbenzimidazolylcarbo-cyanine iodide (JC-1) dye (Invitrogen) that exhibits membrane potential-difference accumulated in the mitochondria. Green fluorescence (~529 nm) emitted from monomer of the dye indicates unstable and low in membrane potential difference, whereas red fluorescence emitted from JC-1 aggregates (~590 nm) shows stable condition with high membrane potential difference.

DNA polymerase γ (POL- γ) activity assay

Mitochondrial fractions (1.2 mg/ml) were lysed in an equal volume of buffer [200 mM NaCl, 50 mM HEPES·KOH (pH 8.0) and 2% Triton X-100] on ice for 20 min, and then centrifuged at 16,000 x g for 10 minutes at 4°C. The supernatant (5 μl) was added to total volume of 50 μl containing 100 mM NaCl, 25 mM HEPES · KOH (pH 8.0), 2.5 mM β-mercaptoethanol, 0.5 mM $MnCl_2$, 0.05 mM aphidicolin, 10 mM deoxythymidine triphosphate (dTTP), 60 μCi/ml of [α-^{32}P]dTTP (3,000 Ci/mmol), 0.1% Triton X-100, 100 μg/ml of acetylated bovine serum albumin, 500 U/ml of RNasin, RNase inhibitor (Promega, Madison, OR, USA), and 50 μg/ml of poly(rA)·oligo(dT)12-18 (GE Healthcare, Piscataway, NJ, USA). The lysate was added to reaction mixture on ice, followed by 20 min incubation in 37 °C water bath, and the reaction was stopped in an ice bath. The aliquots (10 μl) were spotted on nylon transfer membranes (Schleicher and Schuell BioScience GmbH, Dassel, Germany). The paper was washed 3 times in 300 mM NaCl, 30 mM sodium citrate (pH 7.0) for 5 min and once in ethanol, followed by air-dry. Quantification of incorporated dTTP was measured by liquid scintillation counter. Buffer alone and heat inactivation of the lysate in 95 °C were used as negative controls. All assays were carried out in triplicate.
