# Supplementary material for: Mitochondrial nucleoid remodeling and biogenesis are regulated by the p53-p21WAF1-PKCζ pathway in p16INK4a-silenced cells
Source: Aging (Albany NY). 2020 Apr 24;12(8):6700–32. doi: 10.18632/aging.103029 (PMC7202532; doi:10.18632/aging.103029)
Supplement: Supplementary Figures [file aging-12-103029-s003..pdf]

# SUPPLEMENTARY FIGURES

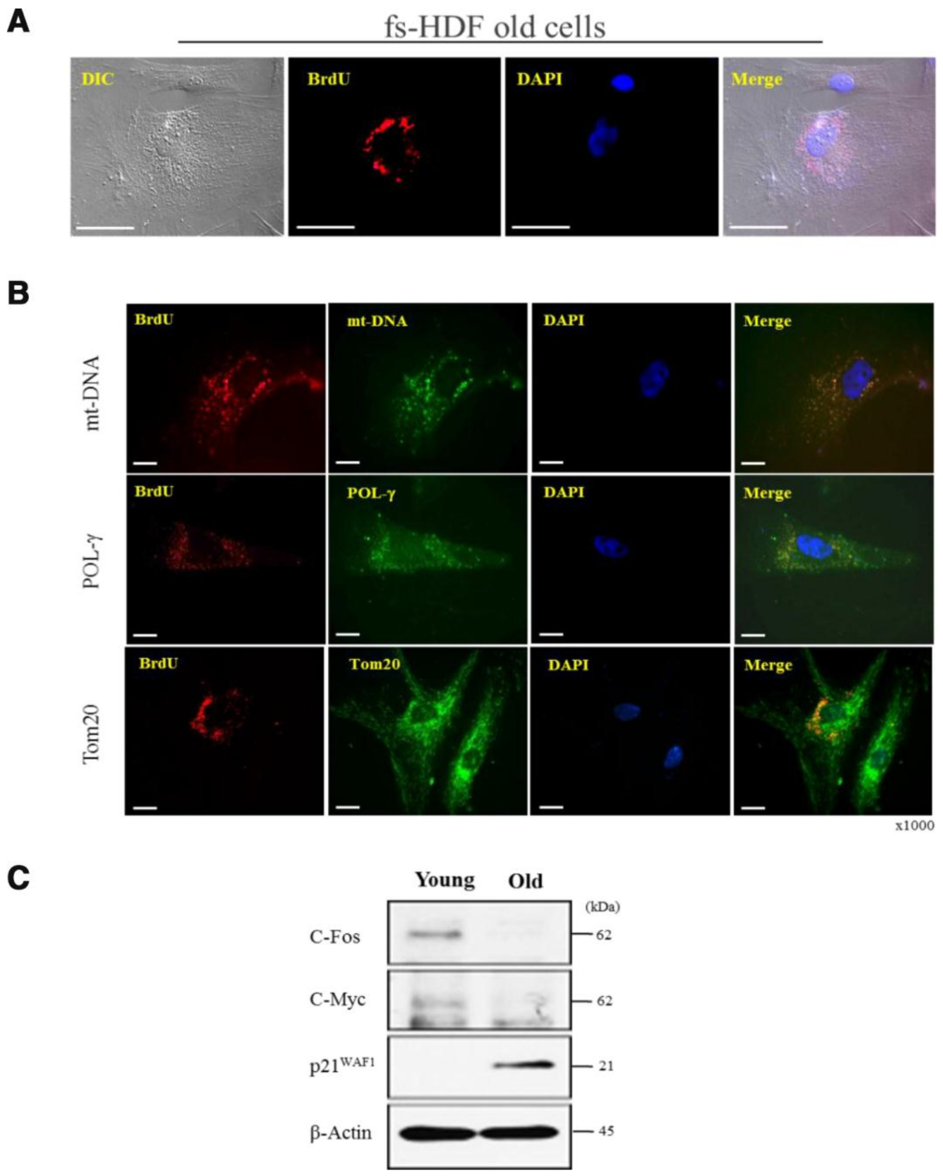


**Supplementary Figure 1. BrdU incorporation into mitochondria, but not into nuclei, of fs-HDF old cells.** (**A**) BrdU (red) and DAPI (blue) fluorescence was merged out of the nucleus of old fs-HDF cell examined by confocal microscopy. Scale bars, 20 µm. (**B**) *First row*: fs-HDF cells stained with anti-BrdU and anti-mtDNA antibodies. DAPI shows blue in nuclei. Scale bars, 10 µm. Note the merge between BrdU incorporation and mt-DNA at the outside of nucleus. *Second row*: Merge of BrdU incorporation and mitochondrial polymerase-ɣ (POL-ɣ) expression at the outside of nucleus. *Third row*: Merge of BrdU incorporation with Tom20 protein (green) at the outside of nucleus. mt-DNA, Pol-ɣ, and Tom20 compose mitochondrial nucleoids. (**C**) Immunoblot analysis of c-Fos, c-Myc and $p21^{WAF1}$ protein expression. β-actin was used as the loading control.

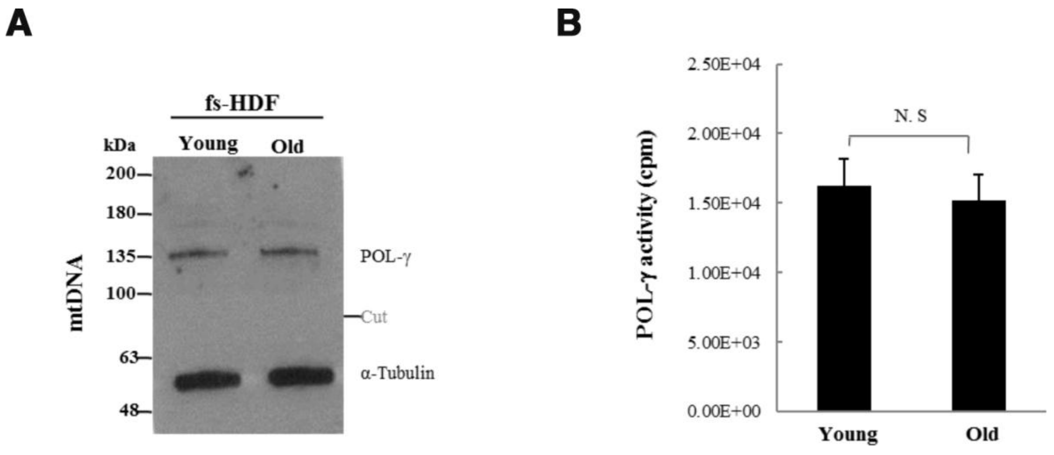


**Supplementary Figure 2. No difference in polymerase-ɣ activity between young and old fs-HDF cells.** (**A**) Expression of POL-ɣ in young (DT; 1 d) and old (DT; 17 d) cells. There is not so different in POL-ɣ expression between young and old cells. (**B**) No difference in the activity of POL-ɣ in replicative senescence of fs-HDF cells. Mitochondrial fractions were isolated from the young and old cells, and then applied to POL-ɣ activity assay for 20 min. All assays were carried out in triplicates and average values were plotted in graph. Statistical analysis was performed using Student´s *t*-test.

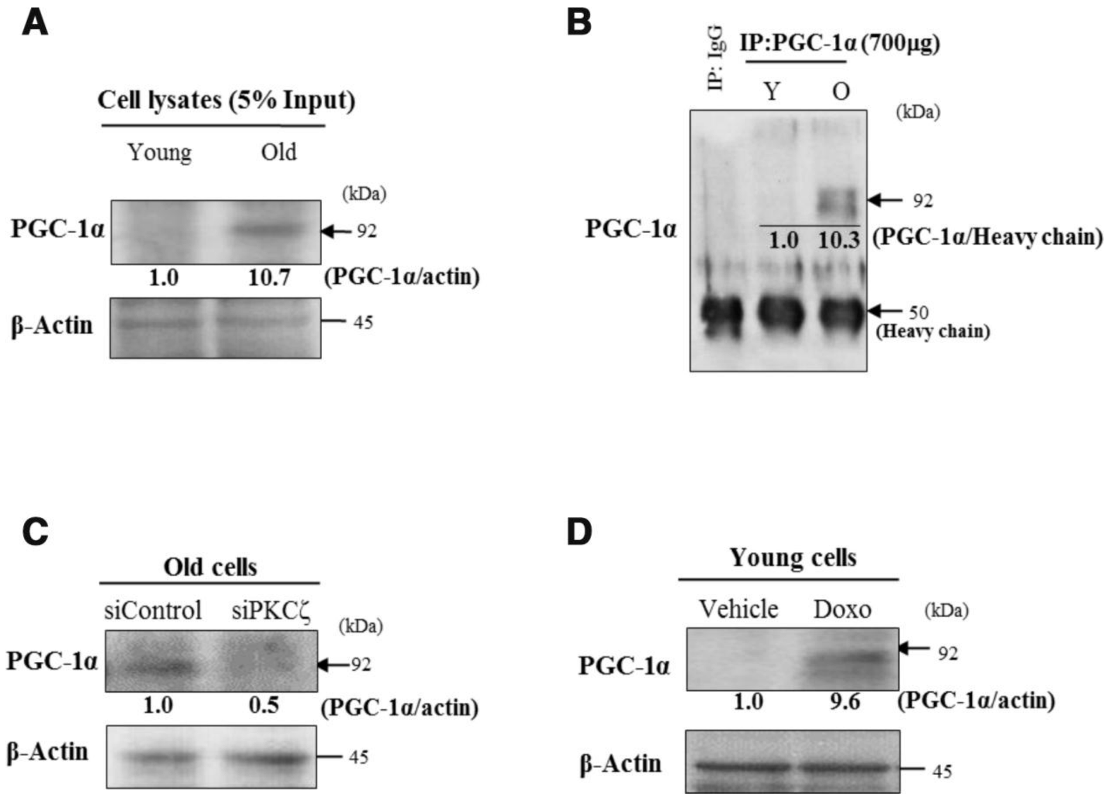


**Supplementary Figure 3. More expression of PGC-1α in old fs-HDF than that in young cells.** (**A**) Slight difference in PGC-1α expression between the young and old cells analyzed by immunoblot (IB) of whole cytoplasm. (**B**) Immunoprecipitation (IP)-IB analysis with anti-PGC-1α antibody. Note higher amount of PGC-1α in the old (O) than young (Y) cells. (**C**) Old fs-HDF cells were transfected with siRNAs-PKCζ, and then expression of PGC-1α was evaluated by immunoblot analysis. (**D**) fs-HDF young cells were treated with vehicle (DMSO) or doxorubicin (Doxo; 100 ng/mL) for 7 days and subjected to immunoblot analysis to measure the expression of PGC-1α. Band intensity was quantified using ImageJ software and normalized to that of β-actin.

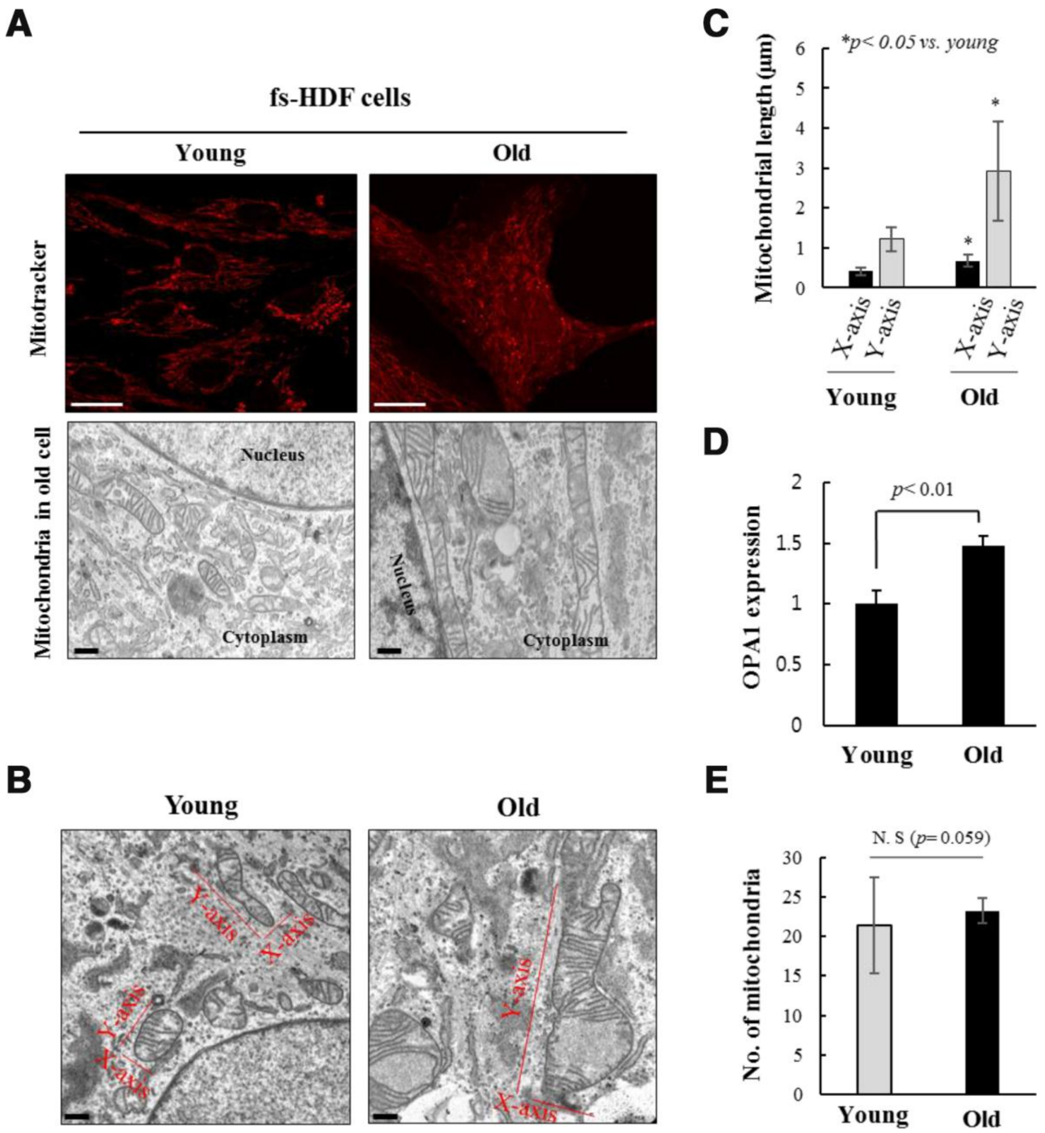
A
fs-HDF cells
Young
Old
Mitotracker
Mitochondria in old cell
Nucleus
Cytoplasm
Nucleus
Cytoplasm
B
Young
Old
Y-axis
X-axis
Y-axis
X-axis
Y-axis
X-axis
C
*p< 0.05 vs. young
Mitochondrial length (μm)
X-axis
Y-axis
X-axis
Y-axis
Young
Old
D
p< 0.01
OPA1 expression
Young
Old
E
N. S (p= 0.059)
No. of mitochondria
Young
Old

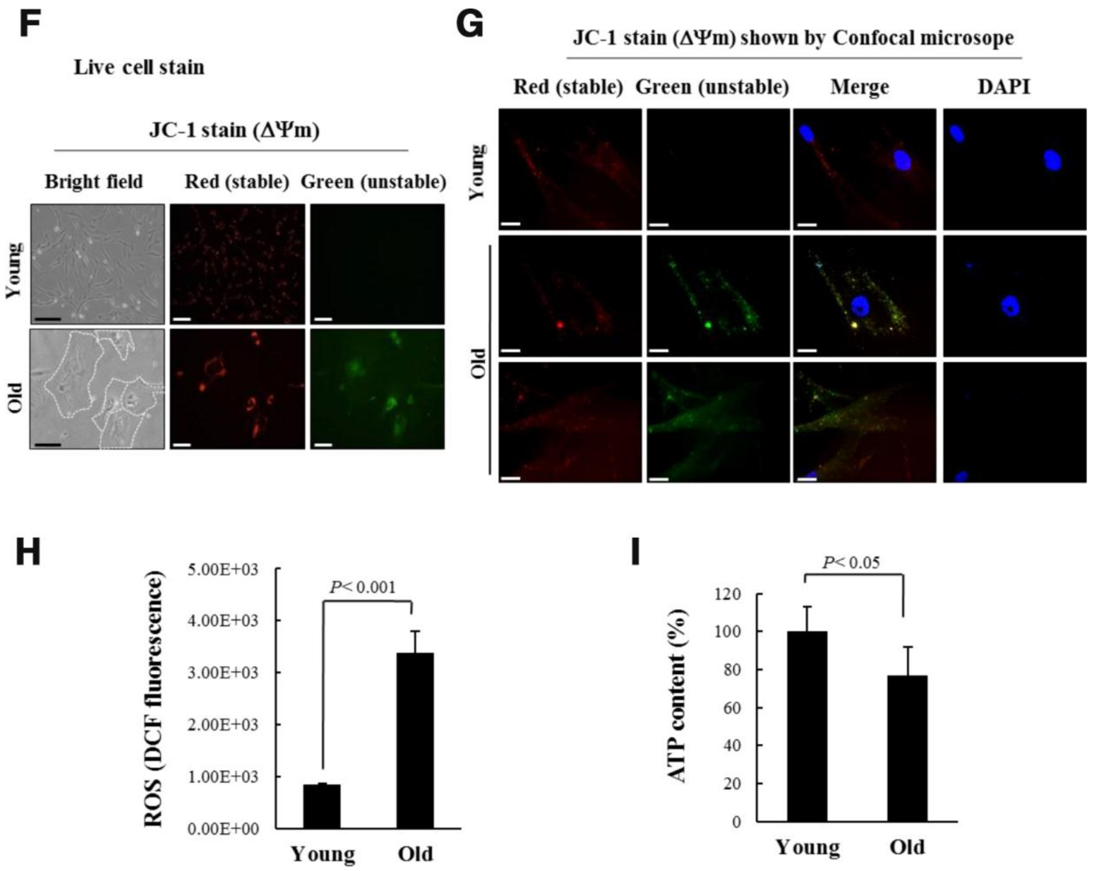


**Supplementary Figure 4. Senescence phenotypes in old fs-HDF cells compared to young cells.** (**A**) MitoTracker (25nM) staining observed by confocal microscopy (upper panels) Scale bars, 25 µm. Lower panels show mitochondria observed by electron microscope (EM) in young and old cells. Note long and enlarged mitochondria in old cells as opposed to short and thin morphology in young cells. Scale bars, 0.6 µm. (**B**) Representative images of mitochondria in old cells under EM. Both x and y axis were marked in the images and the significant increase of the length in the old cells than the young cells was measured (**C**). Scale bars, 0.6 µm. (**D**) RT-qPCR analysis showing the increased expression of OPA1 in old cells. (**E**) There is no significant difference in the numbers of mitochondria between the young and old cells. The variables were quantified using Image J software (n=10 images/group). (**F**) Live cell staining of young and old cells with JC-1 dye to visualize mitochondrial membrane potential differences (ΔΨm) under immunofluorescence microscope. Note absence of green fluorescence in young cells, whereas old cells expressed both red and green fluorescence, indicating loss of ΔΨm in old cells. The enlarged old cell periphery is marked with white dots. Scale bars, 100 µm (black bar) or 20 µm (white bar). (**G**) Confocal microscopic findings of the JC-1 staining. Note absence of green fluorescence in the merged view of young cells, whereas the red and green fluorescence was merged at the outside of nucleus in old cells. Scale bars, 10 µm. (**H**) ROS measurement by flow cytometry analysis. Young and old cells were treated with DCFDA (10µM) for 30 min to determine ROS level by FACS. Note significant increase in old cells. (**I**) ATP content was measured in young and old cells using an ATP determination kit according to the manufacturer’s instruction. ATP levels of the samples were measured based on the standard curve. Data are presented as means ± *S.D.* after 3 independent experiments per group. Statistical analysis was applied by Student´s *t*-test.

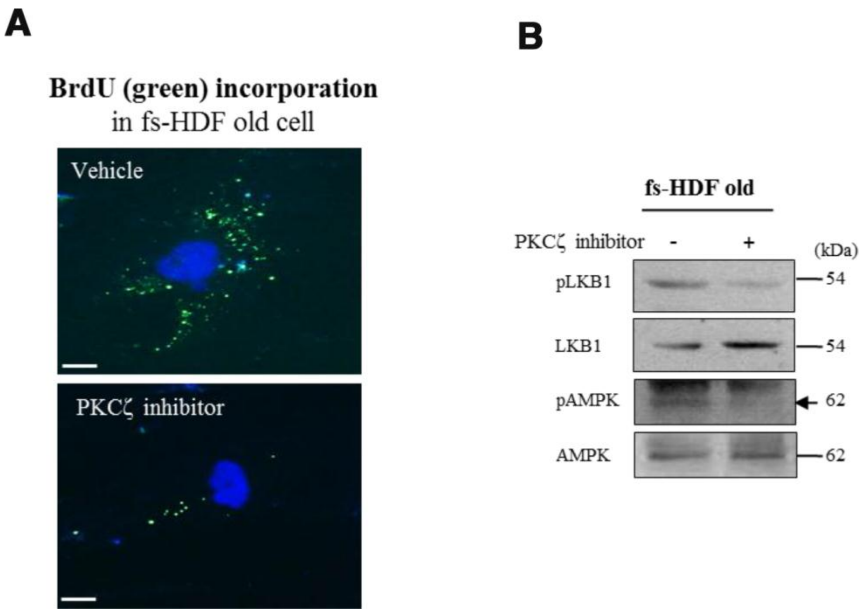


**Supplementary Figure 5. Regulation of mitochondrial nucleoid remodeling by PKCζ signal.** (**A**) Confocal microscopic finding of BrdU incorporation in mitochondria; Treatment of old cells with vehicle (sterilized water) expressed anti-BrdU fluorescence in mitochondria (green), whereas PKCζ inhibitor (10μM of pseudosubstrate peptide, SIYRRGARRWRKL) treatment for 1 h significantly reduced anti-BrdU fluorescence. DAPI (blue) shows the nucleus. Scale bars, 10 μm. (**B**) Immunoblot data exhibiting loss of pLKB1 and pAMPK in old fs-HDF cells treated with PKCζ inhibitor, despite the high levels of LKB1 and AMPK expression.

A

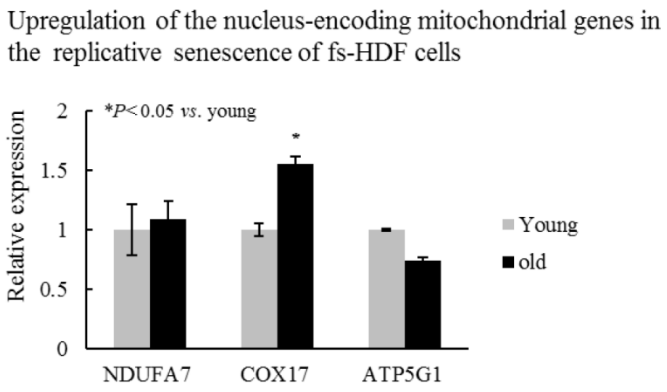


B

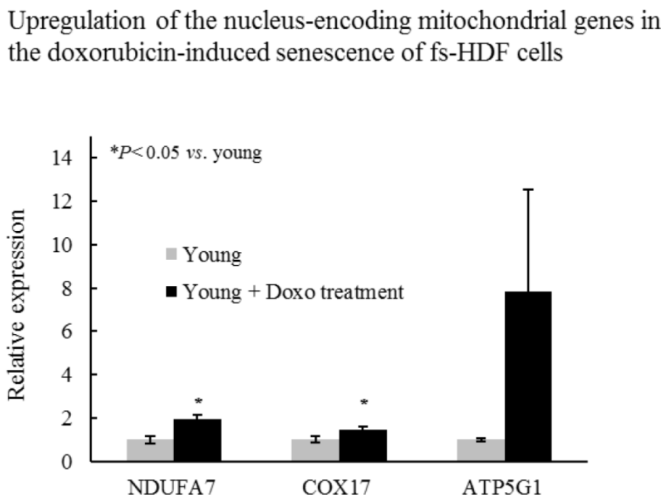


**Supplementary Figure 6. Upregulation of mitochondrial OXPHOS complex in both replicative senescence and doxorubicin-induced senescence of fs-HDF cells.** (**A**) RT-qPCR analysis revealing higher expression of complex-IV subunit in old cells compared to young cells. (**B**) The same experiment was repeated in young cells with or without Doxo-treatment for 8 days, and the cells were subjected to RT-qPCR analysis. Note significant induction of complex-I and -IV subunit gene expression in the Doxo-induced senescence. $^{*}p<0.05$. Data are presented as means ± *S.D.* after 3 independent experiments per group. Statistical analysis was performed using the Student´s *t*-test or one-way ANOVA followed by Tukey's HSD *post hoc* test.

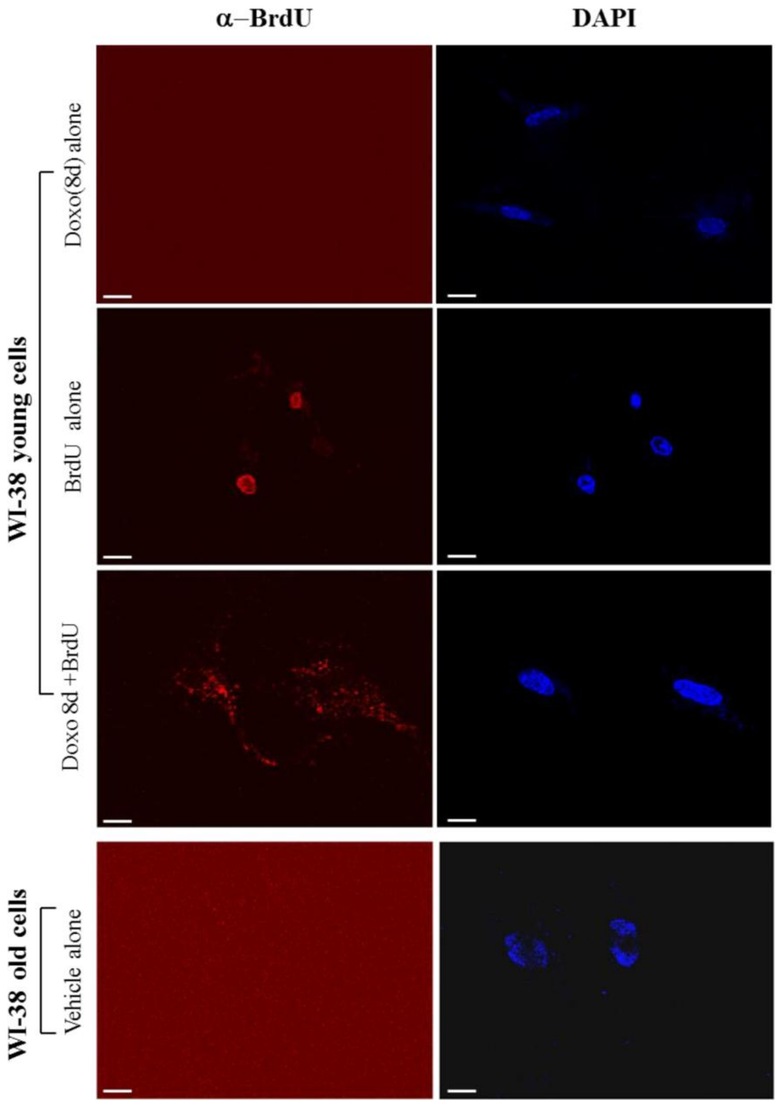


**Supplementary Figure 7. Induction of mitochondrial nucleoid remodeling in WI-38 cells by the doxorubicin treatment.** To confirm the signals regulating mitochondrial nucleoid remodeling *via* the p53-p21$^{WAF1}$ pathway, WI-38 young cells were treated with Doxo for 8 days and then anti-BrdU incorporation was examined by immunocytochemistry; young cells expressed BrdU incorporation in the nuclei (the 2$^{nd}$ row in 1$^{st}$ column), whereas DNA damage induced by Doxo treatment changed BrdU incorporation from nuclei to mitochondria (the 3$^{rd}$ row in 1$^{st}$ column). However, young and old WI-38 cells cannot induce BrdU incorporation neither in nuclei nor in mitochondria without Doxo-treatment (the 1$^{st}$ and the 4$^{th}$ rows in 1$^{st}$ column). Scale bars, 10 µm. Data involve the role of p53-p21$^{WAF1}$ pathway in mitochondrial remodeling in WI-38 senescence.

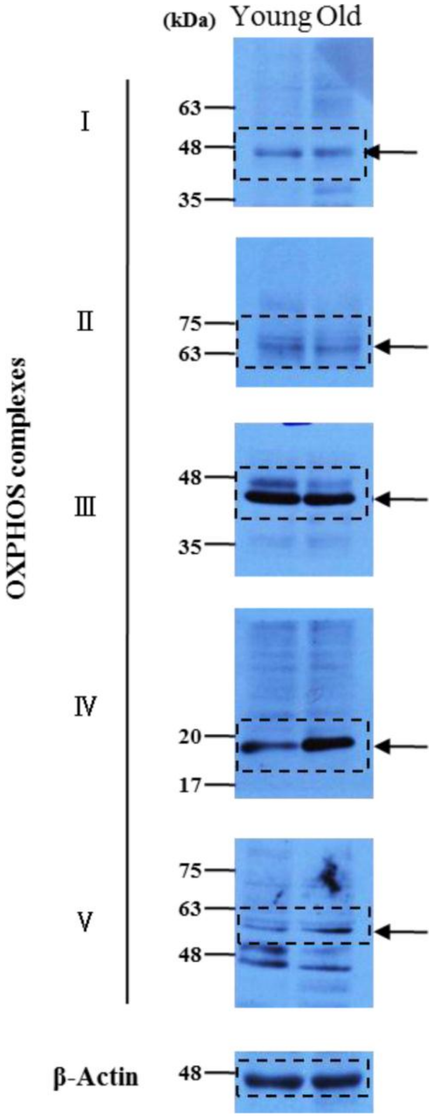
(kDa)
Young
Old
OXPHOS complexes
I
63
48
35
II
75
63
III
48
35
IV
20
17
V
75
63
48
β-Actin
48

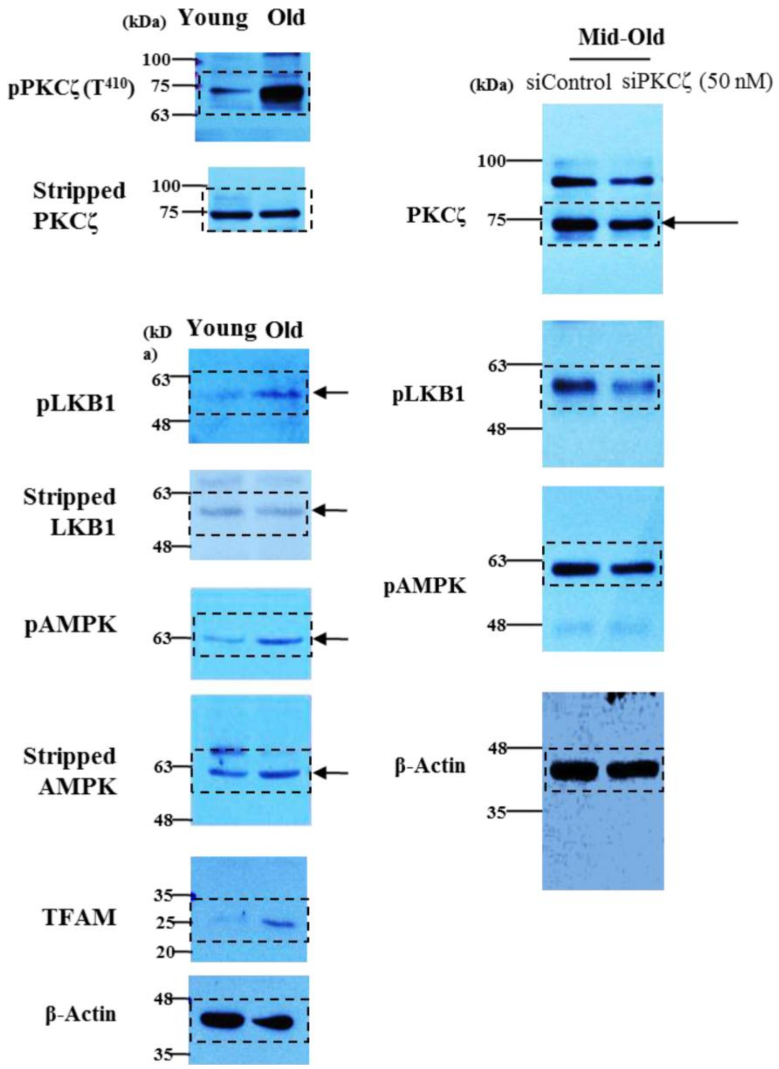

(kDa) Young Old
pPKCζ (T410)
100
75
63
Stripped PKCζ
100
75
Mid-Old
(kDa) siControl siPKCζ (50 nM)
100
PKCζ
75
(kD a) Young Old
pLKB1
63
48
Stripped LKB1
63
48
pAMPK
63
Stripped AMPK
63
48
TFAM
35
25
20
β-Actin
48
35
pLKB1
63
48
pAMPK
63
48
β-Actin
48
35

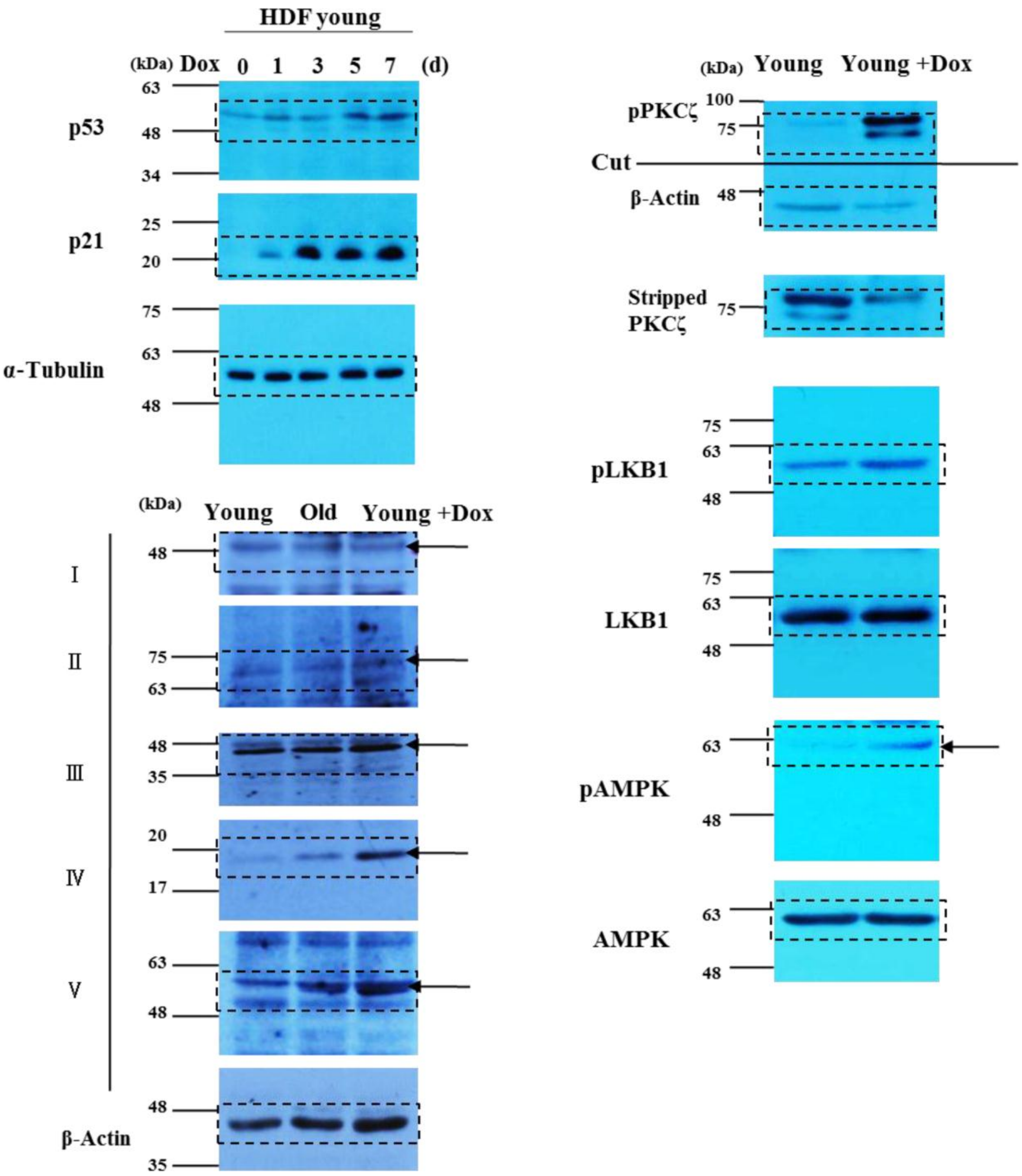
HDF young
(kDa) Dox 0 1 3 5 7 (d)
p53
p21
α-Tubulin
63
48
34
25
20
75
(kDa) Young Old Young +Dox
I
II
III
IV
V
β-Actin
17
35
(kDa) Young Young +Dox
pPKCζ
100
Cut
Stripped
PKCζ
pLKB1
LKB1
pAMPK
AMPK

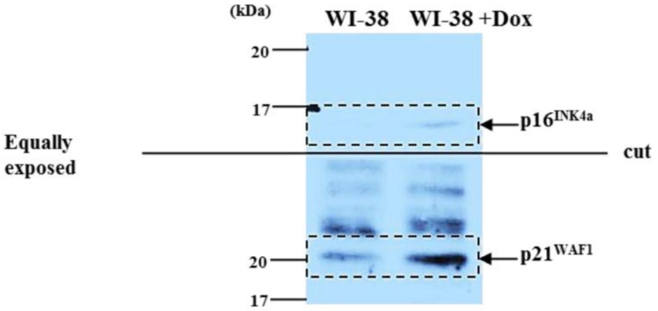
(kDa)
WI-38
WI-38 +Dox
20
17
p16INK4a
Equally
exposed
cut
p21WAF1
20
17

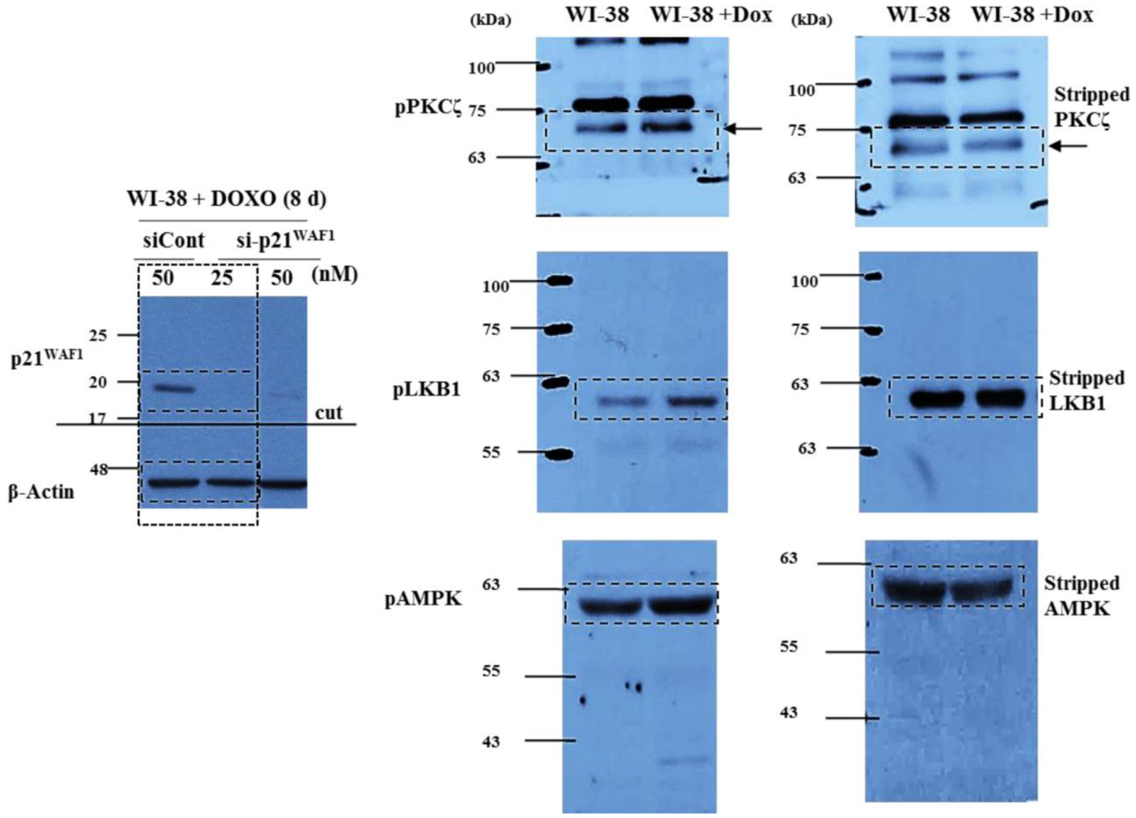
(kDa)
WI-38
WI-38 +Dox
pPKCζ
100
75
63
Stripped
PKCζ
WI-38 + DOXO (8 d)
siCont
si-p21WAF1
50
25
50
(nM)
p21WAF1
25
20
17
cut
48
β-Actin
pLKB1
55
Stripped
LKB1
pAMPK
43
Stripped
AMPK

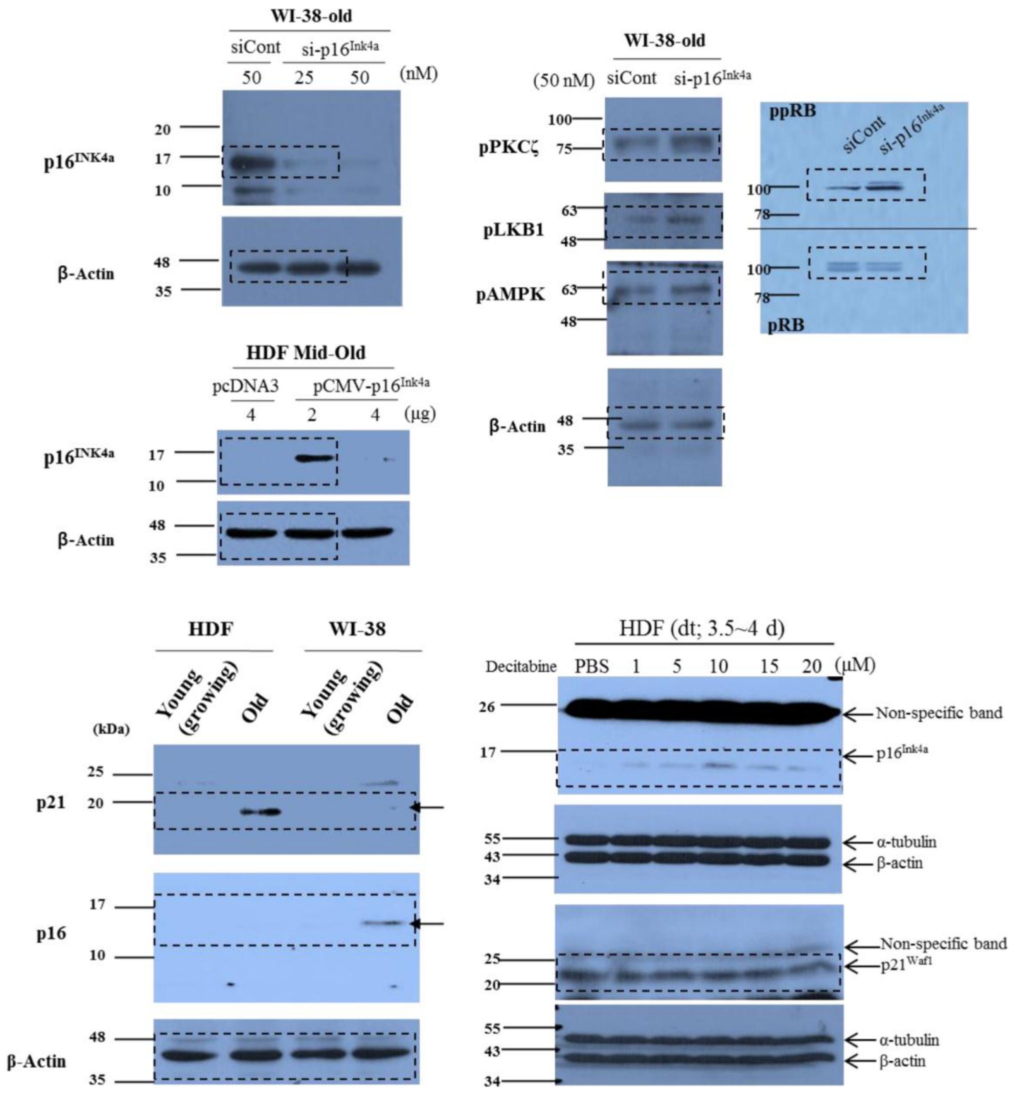


**Supplementary Figure 8. The original film images of immunoblot analysis.** To minimize antibody loss and to allow synchronous detection of more than one protein on the same gel, membranes have been cut with a knife at the target protein molecular weights. Only stripes were further processed, and this explains why figures look incomplete.
