# Supplementary material for: Mitochondrial nucleoid remodeling and biogenesis are regulated by the p53-p21WAF1-PKCζ pathway in p16INK4a-silenced cells
Source: Aging (Albany NY). 2020 Apr 24;12(8):6700–32. doi: 10.18632/aging.103029 (PMC7202532; doi:10.18632/aging.103029)
Supplement: Supplementary Table 1 [file aging-12-103029-s002..pdf]

SUPPLEMENTARY TABLE

Supplementary Table 1. Real-time PCR primer list.

Primer sequences	
COX17 F	5′-CGAGGCTGGCATAGATTTGG-3′
C0X17 R	5′-AACCAGACCCGGCATCTTTC-3′
ATP5G1 F	5′-GCTGAGACCAAGGGCTAAAG-3′
ATP5G1 R	5′-CGGATCAGAGCIGGAGAAATG-3′
NDUFA7 F	5′-CTGTGGGTCCTAGCCACAAG-3′
NDUFA7 R	5′-GCCTTCTGCGACGACATGAT-3′
C0X1 F	5′-CTCCTACTCCTGCTCGCATC-3′
COX1 R	5′-GGGTGACCGAAAAATCAGAA-3′
mtXD4 F	5′-TAGCAGAGACCAACCGAACC-3′
mtXD4 R	5′-GGGGAATGCTGGAGATTGTA-3′
ATPase 8 F	5′-AATATTAAACACAAACTACCACCTACC-3′
ATPase 8 R	5′-TGGTTCTCAGGGTTTGTTATA-3′
PGC-lα F	5′-GGCAGAAGGCAATTGAAGAG-3′
PGC-lα R	5′-TCAAAACGGTCCCTCAGTTC-3′
TFAM F	5′-CCGAGGTGGTTTTCATCTGT-3′
IF AM R	5GCATCTGGGTTCTGAGCTTT-3′
NRF1 F	5′-CCACGTTACAGGGAGGTGAG-3′
NRF1 R	5′-TGTAGCTCCCTGCTGCATCT-3′
c-fos F	5′-TGACTGATACACTCCAAGCGGA-3
c-fos R	5′-CAGGTCATCAGGGATCTTGCA-3′
c-myc F	5′-GATICICTGCTCTCCTCGAC-35
c-myc R	S′-TCCAGACTCIGACCTUTGC-S′
OPA1 F	5′-TGTGATTGAAAACATCTACCTTCCA-3′
OPA1 R	5′-TTTAAGCTTGATATCCACTGTGGTGT-3′
btg2 F	5′-GAAAAGCCGTCCAAGGGC-3′
btg2 R	5′-CTTGTGGTTGATGCGAATGC-3′
pl6 F	5′-TTCCTGGACACGCTGGT-3′
pl6 R	5′-CAATCGGGGATGTCTGAG-31
p21 F	5′-CGACTGTGATGCGCTAATGG-3′
p21 R	S′-CCGTTTTCGACCCTGAGAG-S′
18SrRNA F	5′-GGAGAGGGAGCCTGAGAAAC-3′
18SrRNA R	5′-TCGGGAGTGGGTAATTTGC-3′
NADH dehydrogenase subunit 1 (ND1) F	S′-CCCTAAAACCCGCCACATCT-3′
NADH dehydrogenase subunit 1 (NDl) R	5′-GAGCGATGGTGAGAGCTAAGGT-3′
